# SAMHD1 restrains aberrant nucleotide insertions at repair junctions generated by DNA end joining

**DOI:** 10.1093/nar/gkab051

**Published:** 2021-02-16

**Authors:** Ekaterina Akimova, Franz Josef Gassner, Maria Schubert, Stefan Rebhandl, Claudia Arzt, Stefanie Rauscher, Vanessa Tober, Nadja Zaborsky, Richard Greil, Roland Geisberger

**Affiliations:** Department of Internal Medicine III with Haematology, Medical Oncology, Haemostaseology, Infectiology and Rheumatology, Oncologic Center, Paracelsus Medical University, Salzburg, Austria; Salzburg Cancer Research Institute - Laboratory for Immunological and Molecular Cancer Research (SCRI-LIMCR); Cancer Cluster Salzburg, 5020 Salzburg, Austria; Department of Biosciences, Paris Lodron University of Salzburg, 5020 Salzburg, Austria; Department of Internal Medicine III with Haematology, Medical Oncology, Haemostaseology, Infectiology and Rheumatology, Oncologic Center, Paracelsus Medical University, Salzburg, Austria; Salzburg Cancer Research Institute - Laboratory for Immunological and Molecular Cancer Research (SCRI-LIMCR); Cancer Cluster Salzburg, 5020 Salzburg, Austria; Department of Internal Medicine III with Haematology, Medical Oncology, Haemostaseology, Infectiology and Rheumatology, Oncologic Center, Paracelsus Medical University, Salzburg, Austria; Salzburg Cancer Research Institute - Laboratory for Immunological and Molecular Cancer Research (SCRI-LIMCR); Cancer Cluster Salzburg, 5020 Salzburg, Austria; Department of Internal Medicine III with Haematology, Medical Oncology, Haemostaseology, Infectiology and Rheumatology, Oncologic Center, Paracelsus Medical University, Salzburg, Austria; Salzburg Cancer Research Institute - Laboratory for Immunological and Molecular Cancer Research (SCRI-LIMCR); Cancer Cluster Salzburg, 5020 Salzburg, Austria; Department of Internal Medicine III with Haematology, Medical Oncology, Haemostaseology, Infectiology and Rheumatology, Oncologic Center, Paracelsus Medical University, Salzburg, Austria; Salzburg Cancer Research Institute - Laboratory for Immunological and Molecular Cancer Research (SCRI-LIMCR); Cancer Cluster Salzburg, 5020 Salzburg, Austria; Department of Internal Medicine III with Haematology, Medical Oncology, Haemostaseology, Infectiology and Rheumatology, Oncologic Center, Paracelsus Medical University, Salzburg, Austria; Salzburg Cancer Research Institute - Laboratory for Immunological and Molecular Cancer Research (SCRI-LIMCR); Cancer Cluster Salzburg, 5020 Salzburg, Austria; Department of Biosciences, Paris Lodron University of Salzburg, 5020 Salzburg, Austria; Department of Internal Medicine III with Haematology, Medical Oncology, Haemostaseology, Infectiology and Rheumatology, Oncologic Center, Paracelsus Medical University, Salzburg, Austria; Salzburg Cancer Research Institute - Laboratory for Immunological and Molecular Cancer Research (SCRI-LIMCR); Cancer Cluster Salzburg, 5020 Salzburg, Austria; Department of Biosciences, Paris Lodron University of Salzburg, 5020 Salzburg, Austria; Department of Internal Medicine III with Haematology, Medical Oncology, Haemostaseology, Infectiology and Rheumatology, Oncologic Center, Paracelsus Medical University, Salzburg, Austria; Salzburg Cancer Research Institute - Laboratory for Immunological and Molecular Cancer Research (SCRI-LIMCR); Cancer Cluster Salzburg, 5020 Salzburg, Austria; Department of Internal Medicine III with Haematology, Medical Oncology, Haemostaseology, Infectiology and Rheumatology, Oncologic Center, Paracelsus Medical University, Salzburg, Austria; Salzburg Cancer Research Institute - Laboratory for Immunological and Molecular Cancer Research (SCRI-LIMCR); Cancer Cluster Salzburg, 5020 Salzburg, Austria; Department of Internal Medicine III with Haematology, Medical Oncology, Haemostaseology, Infectiology and Rheumatology, Oncologic Center, Paracelsus Medical University, Salzburg, Austria; Salzburg Cancer Research Institute - Laboratory for Immunological and Molecular Cancer Research (SCRI-LIMCR); Cancer Cluster Salzburg, 5020 Salzburg, Austria

## Abstract

Aberrant end joining of DNA double strand breaks leads to chromosomal rearrangements and to insertion of nuclear or mitochondrial DNA into breakpoints, which is commonly observed in cancer cells and constitutes a major threat to genome integrity. However, the mechanisms that are causative for these insertions are largely unknown. By monitoring end joining of different linear DNA substrates introduced into HEK293 cells, as well as by examining end joining of CRISPR/Cas9 induced DNA breaks in HEK293 and HeLa cells, we provide evidence that the dNTPase activity of SAMHD1 impedes aberrant DNA resynthesis at DNA breaks during DNA end joining. Hence, SAMHD1 expression or low intracellular dNTP levels lead to shorter repair joints and impede insertion of distant DNA regions prior end repair. Our results reveal a novel role for SAMHD1 in DNA end joining and provide new insights into how loss of SAMHD1 may contribute to genome instability and cancer development.

## Graphical Abstract

**Graphical Abstract:**
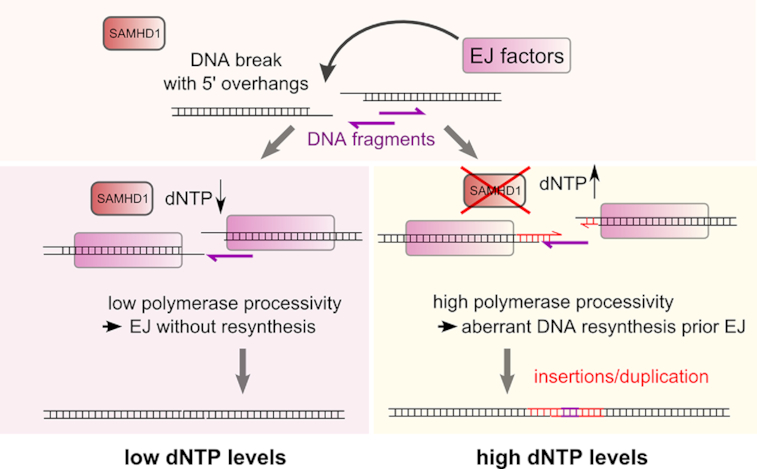
The dNTPase SAMHD1 keeps dNTP levels at moderate levels, incompatible with aberrant overhang resynthesis and insertions at DNA repair joints.

## INTRODUCTION

DNA double strand breaks (DSBs) are the most severe form of DNA damage and require a quick and proper response by the DNA repair machinery to avoid the occurrence of structural chromosomal rearrangements by illegitimate end joining of distant DNA ends. Any perturbations of the DNA DSB repair pathway are contributing to genetic instability, the acquisition of mutations and chromosomal aberrations, which in turn facilitate the development of cancer (1). Generally, DNA DSBs are fixed by diverse repair pathways, depending on the availability of homologous DNA regions for template-guided repair. Presence of homologous DNA regions stimulates DNA repair by homologous recombination, whereas in G1 phase of the cell cycle, where no homologous sister chromatids are present, DNA DSBs can only be repaired by non-homologous end joining (NHEJ) (2–4). However, while homologous repair is thus largely restricted to S/G2 phase, NHEJ is still the predominant repair pathway throughout the cell cycle (5,6). While DNA ends with perfectly matching cohesive overhangs can simply be re-ligated by the ligase IV complex (7), repair of non-cohesive ends relies on a set of specialized enzymes mediating joining with or without prior resection, blunting or extension of DNA ends, which can be assigned to classical or alternative NHEJ (c-NHEJ or a-NHEJ) (2). Both NHEJ pathways prefer the usage of short patches of microhomology (≥1 nt) within DNA ends for end joining, with a-NHEJ typically depending on longer regions of homology between the two DNA ends (>4 nt). Thus, a-NHEJ is also termed microhomology-mediated end joining (MMEJ) (4).

SAMHD1 (SAM domain and HD domain-containing protein 1) was initially discovered as protein with deoxynucleoside triphosphate triphosphohydrolase (dNTPase) activity and putative nuclease activity. It was reported that SAMHD1 is mainly a restriction factor for retroviruses and retroviral elements by reducing the pool of available dNTPs to a level incompatible with reverse transcription of the virus genome (8–12). In addition, SAMHD1 protein was found to be associated with foci of DNA double strand breaks (DSBs), proposing a possible role for SAMHD1 in DNA DSB repair (13). Indeed, it was shown that SAMHD1 promotes DSB repair by homologous recombination independent from its dNTPase activity but dependent on its interaction with the endonuclease CtIP. In this model, CtIP is recruited to DSBs via interaction with SAMHD1, thus resecting DNA ends to generate 3′ overhangs, which facilitate strand invasion for homologous recombination (14).

In this study, we asked whether aside of involvement in homologous DNA repair, SAMHD1 may have an additional effect on NHEJ. Our own results reveal a novel role of SAMHD1 for DNA DSB repair by NHEJ and give new insights into its implication in genome stability and cancer development, and corroborate a very recent report on a novel role of SAMHD1 in regulating class switch recombination in B lymphocytes (15).

## MATERIALS AND METHODS

### Cell lines and transfection

HEK293 and HeLa cell lines were cultured in RPMI medium supplemented with 10% FBS, 1% l-glutamine and 1% Pen/Strep. For transient transfections, GeneJuice Transfection Reagent (Novagen) was used.

### Plasmid based DNA repair assay

Repair assays on linearized plasmids were performed according to our recent publication (16). In brief, HEK293 cells were either non-transfected or transfected with an expression construct encoding human SAMHD1-Flag (Sinobiological) or mutants using GeneJuice (Novagen). One day post transfection, a respective pool of linear plasmids (pool 1: substate #1–#9; pool 2: substrate #11–#18; sequences provided in Supporting Table S1) was transfected and total DNA was extracted from cells 72 h post transfection of the plasmid pool (DNeasy Blood & Tissue Kit, Qiagen). Repaired plasmid-junctions were PCR-amplified using specific primers with different 5′ tags specific for each sample. Amplicons were pooled and sequenced on the Illumina MiSeq platform (MWG eurofins).The unique 6-bp region downstream of the conserved primer-binding sites from each plasmid was used for assigning relative frequencies of the individual plasmid substrates PCR-amplified from the plasmid pool. Analyses of read length, frequency, and sequence were performed using custom BASH and PERL scripts as previously described (16).

### RT-PCR, western blotting and microscopy

Total RNA was extracted from HEK293 cells (RNeasy Mini Kit, Qiagen) and subjected to first strand cDNA synthesis (iScript). RT-PCR was performed on first strand cDNA using specific primers for endogenous SAMHD1 and transgenic SAMHD1. Western blots were performed on cell lysates using antibodies specific for SAMHD1 (ab67820, Abcam) and Flag-tag (clone M2, Sigma).

For the subcellular localization analysis, HEK293*^SAMHD1-KO^*cells were transferred onto poly-l-lysine coated microscopy slides 4 hours after transfection with mCherry-SAMHD1 fusion constructs and nCas9 constructs expressing zsGreen1 from a separate promoter. Twenty four hours later, the cells were fixed with 4% PFA and stained with anti-fade reagent with DAPI solution (DAKO). Microscopy was performed on an Olympus IX81 using 60× magnification, DAPI, FITC and Cy3 channels for DAPI, ZsGreen1 and mCherry detection.

### Measurement of cellular dNTP levels

Cellular dNTPs were quantified as recently described elsewhere (17). In brief, HEK293 SAMHD1-KO cells were transiently transfected with 1 μg of SAMHD1 variant (wild type, K11A, K312A or K484T) using GeneJuice Transfection Reagent (Novagen) or cells (HEK293, HEK293 SAMHD1-KO and HeLa) were treated with DMSO or 2.5 mM dNs (Sigma Aldrich). Forty-eight hours post transfection/treatment the cells were harvested in ice-cold PBS, counted and the same cell number was aliquoted for all samples. The harvested cells were resuspended in 60% ice-cold methanol, denatured at 95°C for 3 min and centrifuged at 18 500 ×g for 6 min at 4°C. Subsequently, the supernatants were transferred into new tubes and evaporated in Eppendorf Concentrator 5301 for 1 h at 60°C. dNTP extracts were resuspended in 100 μl ice-cold nuclease-free water. The extracts were stored at –80°C up to 1 week. The extracts were subjected to a Q5 DNA polymerase and EvaGreen-based assay for dNTPs using dNTP-specific 50-nt templates with conserved primer binding sites as described in (17). The baseline and the end-point fluorescence were read above the melting temperature of RNAse HII-nicked DNA as determined according to melt curve analyses: 75°C for dATP and dCTP, 78°C for dTTP and 73.5°C for dGTP. The higher the concentration of available dNTPs, the higher is the amount of dsDNA synthesized from the 50 nt template and the higher is the fluorescence, measured on a ViiA7 Real-Time PCR system (ThermoFisher).

### CRISPR/Cas9 constructs and cloning

SAMHD1 mutants were cloned using site directed mutagenesis with standard protocols using In-Fusion HD cloning (Takarabio). mCherry fusion constructs were generated by cloning RG1275 (5′- CCGCCACCAAGCTTG gccaccATGGTGAGCAAGGG-3′) and RG1276 (5′- tcggctcgctgcatG TACTTGTACAGCTCGTCCATGCCG-3′) amplified mCherry into KpnI linearized SAMHD1 constructs using In-Fusion HD cloning (Takarabio).

HEK293*^SAMHD1-KO^* cells (knockout of SAMHD1 in HEK293 cells) were generated by cloning annealed oligos RG1075 5′-CCGGGTCATCGCAACGGGGACGCT-3′ and RG1076 5′-AAACAGCGTCCCCGTTGCGATGAC-3′ (guide RNA for exon 1 of *SAMHD1*) into pGuide-it-ZsGreen1 Vector (Clontech) and transient transfection of HEK293 cells using GeneJuice (Novagen). Three days post transfection, Zsgreen1 positive single cells were sorted into 96-well plates (FACS ARIAIII, Beckton Dickinson). Two weeks post sort, colonies were expanded, genomic DNA was isolated (Qiagen), Cas9 target region was PCR amplified using Phusion polymerase (ThermoFisher) and primers RG1105 (5′-CTACCTCGGATGTTCTTCAGCAG-3′) and RG1106 (5′-AATAGGCTGCCAATACTCCTTGG-3′) and sequenced. Knockout clones were expanded and several stocks frozen for further experiments.

For induction of chromosomal deletion on chr11q, two pairs of nCas9 constructs were generated using cloning of the following annealed oligos into ZsGreen1 Vector (Clontech): RG1195 5′-CCGGGGCTTGTGCCCTTCCCTTCA-3′ and RG1196 5′-AAACTGAAGGGAAGGGCACAAGCC-3′; RG1197 5′-CCGGCTGCTGACTGAAGAGCCTTC-3′ and RG1198 5′-AAACGAAGGCTCTTCAGTCAGCAG-3′; RG1199 5′-CCGGTTTCACCATGTTGCCCAGGC-3′ and RG1200 5′-AAACGCCTGGGCAACATGGTGAAA-3′; RG1201 5′-CCGGACAAAACTTAGCTGGGCGTG-3′ and RG1202 5′-AAACCACGCCCAGCTAAGTTTTGT-3′. Cas9 was mutated to nCas9 by generating D10A variants (18) of these vectors by PCR amplifying a 786 bp fragment using Phusion polymerase (ThermoFisher) and primers RG1191 (5′-CCATGGTGGCGAATTCTCCAGGCGATCTGACGG-3′) and RG1192 (5′-CAGCCCACGCTGTTGGTACCGATGGCCAG-3′) and cloning this fragment into KpnI/EcoRI (ThermoFisher) digested vectors using HD-in fusion cloning (Clontech).

### nCas9-based DNA repair assay

For nCas9-based DNA repair assay HEK293, HEK293*^SAMHD1-KO^* and HeLa cells were transiently transfected with four del11q inducing nCas9 constructs using GeneJuice Transfection Reagent (Novagen), 250 ng each, according to manufacturer's protocol. Simultaneously, the cells were co-transfected with 1 μg of SAMHD1 variant (wild type, K11A, K312A or K484T). Forty-eight hours post transfection the cells were harvested and DNA was isolated using DNeasy Blood & Tissue Kit (Qiagen). The del11q junctions were PCR amplified using 100ng input DNA and primers RG1209 (5′-TTCAGCCATGGTAGAATACAGCACTAC-3′) and RG1210 (5′-CTGGAGTGCAGTAAACCTAGGAAC-‘3). PCR products were gel excised (Qiagen) and TOPO cloned (Invitrogen) followed by Sanger sequencing of individual clones (eurofins genomics). Inserted DNA was mapped using BLAT search (https://genome.ucsc.edu/cgi-bin/hgBlat). For amplicon sequencing on the in-house MiSeq platform (Illumina), PCR amplified breakpoint junctions from independent experiments (as indicated by n-values) were purified from agarose gels, pooled and re-amplified using primers RG1309 (5′-TCGTCGGCAGCGTCAGATGTGTATAAGAGACAGTTCAGCCATGGTAGAATACAGCACTAC-3′) and RG1310 (5′-GTCTCGTGGGCTCGGAGATGTGTATAAGAGACAGCTGGAGTGCAGTAAACCTAGGAAC-3′) followed by indexing and AMPure XP bead purification (Beckman Coulter). For dN supplementation, cells were transfected with del11q inducing nCas9 constructs. Four hours post transfection, cells were mock-treated with DMSO or treated with 2.5 mM dNs (dA, dT, dC, dG; Sigma-Aldrich) and incubated for further 48 h. To determine contribution of DNA-PKc to del11q, cells were mock-treated (DMSO) or treated with 10μM DNA-PKc inhibitor NU7441 (Selleckchem) at time of transfecting nCas9 constructs. DNA was isolated 24 h post transfection and presence of del11q was determined by PCR using primers RG1209 and RG1210 using 60ng template DNA.

For analysis of PCR-amplified junctions sequenced on the Illumina platform (MiSeq), demultiplexed paired end fastq files were adapter- and quality-trimmed using Trimmomatic (19), v0.33, default settings) and stitched using FLASh (20), v1.2.11, -r 300, -f 350, -s 35). Sequences and length of reads containing exact matches of forward and reverse primer plus five 3′ bases of the chr11 target site were extracted (grep TTCAGCCATGGTAGAATACAGCACTACTTAGAT.*GTGGGAAGGTTCCTAGGTTTACTGCACTCCAG) and used for further analysis. For both, Sanger and amplicon-sequencing, only unique sequences were considered for analysis in order to examine individual del11q junctions. For microhomology analysis, junctions that contain insertions were not included.

To evaluate insertions at the nickase site, the extracted sequences were filtered for unique sequences longer than the expected wild type sequence (≥376nt). These sequences were transformed to fasta format and a search for sequence homologies was performed using NCBI blast+ (v2.10.0) (21), the hg19 human genomic database was completed by adding sequences of used plasmids and the del11q junction sequence. Sequence homologies to the nickase site at chr11 (chr11:106892584-107762754) were excluded. Furthermore, homologies that map to nickase site or del11q junction were selected to define intervals of homologies (alignment ≥87%). All other homologies (alignment ≥ 99%) were checked for overlaps with these intervals. The overlapping parts were then trimmed, whereas the remaining homology sequences (parts that do not overlap with del11q junction) were used for further analysis if their length was ≥15 bp. In the next step, we checked if single reads comprise several homologies that do not overlap with each other. If homologies within the same read did not overlap, both or all of them were further analysed. If homologies overlapped, the one with the highest bit score was selected (i.e. the longest one). All homologies passing this filter were manually re-blasted for alignment to the del11q junction using NCBI BLAST tool (https://blast.ncbi.nlm.nih.gov/Blast.cgi), and del11q junction homologies were excluded. Since some of the sequences produced homologies on multiple loci with equal bit score, one hit was selected randomly, and ambiguous blast-hits of reads were indicated in red within the circus plots.

Moreover, genomic positions of detected homologies were compared to the list of aphidicolin-sensitive genes generated by Crosetto *et al.* (22), which was downloaded from the BLESS supporting website http://breakome.utmb.edu/Home.html.

For statistical analysis of insertions, the numbers of reads containing homologies versus reads without homologies were evaluated by Fisher's exact test.

All analyses were performed on a Linux Redhat system using custom bash or R (v3.6.1) scripting.

The following R-packages were used: BiocManager, BSgenome, BSgenome.Hsapiens.UCSC.hg19, dplyr, IRanges, GenomeRanges, stringr, openxlsx, spgs, Biostrings, ggplot2, EasyGgplot2, devtools, circlize (23).

### Statistics and data availability

No explicit power analysis was used for sample size estimation. *N*-values indicate biological replicates, which were independent experiments (cells handled separately) but sequenced on the Illumina platform as pooled (indexed) samples. All indices are provided together with sequencing data, which have been deposited in the ArrayExpress database at EMBL-EBI (www.ebi.ac.uk/arrayexpress) under accession number E-MTAB-8382.

## RESULTS

### SAMHD1 affects DNA DSB repair by NHEJ

**Figure 1.**
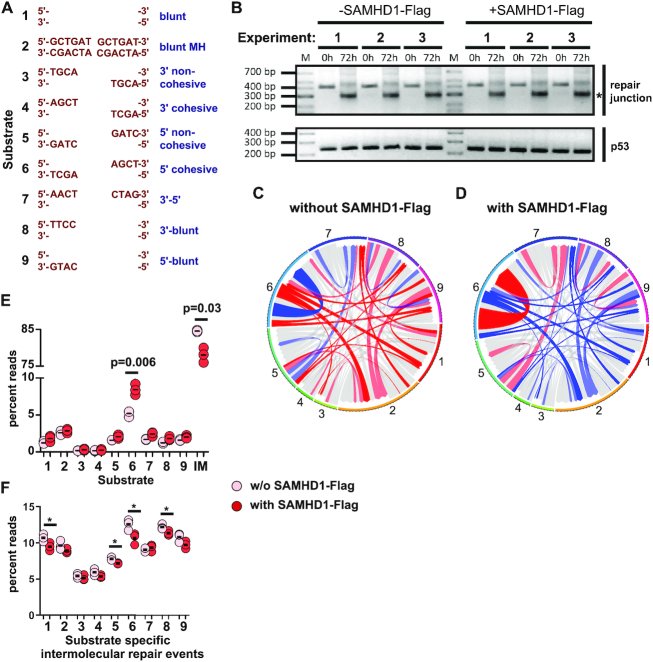
DNA Repair of plasmid substrates is skewed by SAMHD1. (**A**) A pool of linear DNA plasmids (substrate #1-#9, with the respective DNA end structures indicated) was transfected into SAMHD1-Flag expressing or non-expressing HEK293 cells. (**B**) Repaired plasmid junctions were PCR-amplified from extracted DNA 0 and 72 h post transfection from 3 independent experiments. A control PCR on TP53 is shown on the bottom. Bands corresponding in size to repair junctions are indicated with an asterisk. (**C, D**) Repair frequencies of inter- and intramolecular repair events (substrate #1–#9) are shown as circos plots for SAMHD1-Flag non-expressing and expressing HEK293 cells. The size of the segments reflects the occurrence of the nine different DNA plasmids serving as substrate for repair. Repair frequencies are indicated by size of ribbons (mean values from three independent experiments). Start of ribbon denotes forward primed arms; arrow of ribbon denotes reverse primed arm of substrates. Repair junctions significantly overrepresented (C vs D and vice versa) are colored red (*P* < 0.05) and light red (0.05 < *P* < 0.1), and the correspondingly underrepresented repair junctions are colored blue and light blue, respectively. Significances were calculated by two-tailed t-tests with unequal variances, *n* = 3. (**E**) Repair frequencies from C and D are shown separately for intramolecular (#1–#9) and intermolecular (IM) repair in HEK293 cells either expressing or not-expressing SAMHD1-Flag. (**F**) Repair frequencies from C and D are shown separately for intermolecular repair in HEK293 cells either expressing or not-expressing Flag-SAMHD1. Means are indicated; significances were calculated by two-tailed t-tests with unequal variances, *n* = 3.

As previous work suggested involvement of SAMHD1 in response to DNA damage, we aimed to investigate the contribution of SAMHD1 to accurate and efficient DNA DSB repair by NHEJ. To this end, we first wanted to test whether overexpression of SAMHD1 affects *in vivo* joining of artificial DNA substrates. Therefore, we used HEK293 cells, which express low levels of SAMHD1 protein, and transfected them with Flag-tagged SAMHD1 to induce overexpression (Supplementary Figure S1). Twenty-four hours post transfection, mock transfected cells or Flag-SAMHD1 transfected cells were subjected to a previously reported plasmid based DNA DSB repair assay to analyze *in vivo* joining of a pool of different DNA end structures in three independent experiments (16). In this assay, a pool of nine plasmids, which harbor individual 350-bp inserts flanked by conserved primer-binding sites, is digested *in vitro* with different restriction enzymes. This yields nine linearized DNA plasmids with defined DNA break structures ranging from blunt ends with and without a 6-bp microhomology to cohesive and noncohesive 3′ and 5′ protruding ends with 4 nt overhangs (substrate #1 to #9; Figure 1A, Supplementary Table S1). Upon transfection into cells, the linear DNA substrates are recognized by the cellular DNA DSB repair machinery and rejoined. Seventy-two hours post transfection, we isolated DNA from cells and PCR-amplified repair junctions of pooled linear plasmid substrates using the conserved primer binding sites and subjected them to amplicon sequencing (Figure 1B). We then calculated relative repair frequencies of the respective DNA ends by mapping sequence reads to the nine repair substrates as previously described (16). Thereby, we found that SAMHD1 overexpression severely affected the pattern of inter- and intramolecular repair frequencies (Figure 1C–F). Particularly, we found that cohesive rejoining of the compatible ends on substrate #6 occurred more frequently in presence of Flag-SAMHD1 overexpression (Figure 1E). Concurrently, intermolecular repair events between different substrates occurred less frequently in presence of Flag-SAMHD1 overexpression, independent of the end structure (Figure 1E and F).

### SAMHD1 facilitates joining of protruding 5′ DNA ends carrying short regions of complementarity

**Figure 2.**
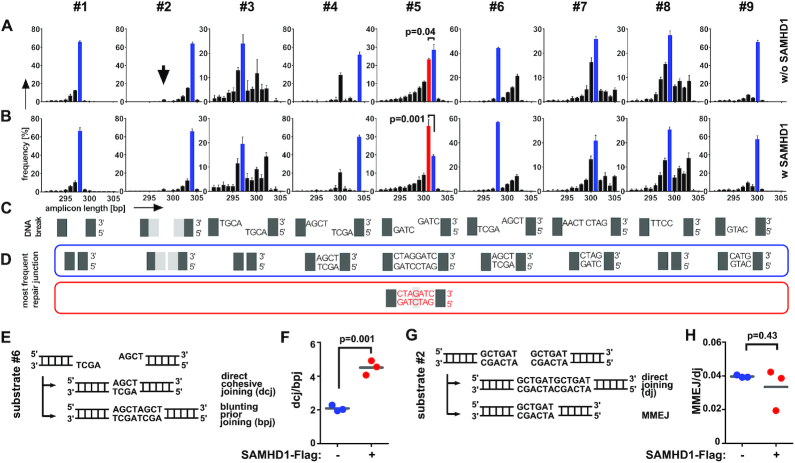
Repair junctions are modulated by SAMHD1. (A, B) PCR-amplified repair junctions from SAMHD1-Flag non-expressing (**A**) or expressing (**B**) HEK293 cells were sequenced and the frequencies of the lengths of the respective sequences are shown for each DNA plasmid substrate (#1-#9). The arrow within the graph for substrate #2 denotes the size of the repair junction corresponding to junctions repaired by MMEJ using a 6bp microhomology. (**C**) The respective DNA break structures for DNA plasmid substrates #1- #9 is shown. (**D**) The sequences of the most frequent junctions for cells not expressing Flag-SAMHD1 are shown and framed in blue. The most frequent repair junctions for Flag-SAMHD1 expressing cells (if different to non-expressing cells), are shown below and framed in red. The color of the frame allocates the sequences to the respective bars shown in (A, B). Bars are showing mean + SD from three independent experiments. (**E**) DNA break structure of DNA plasmid substrate #6 carrying cohesive 4 nt 5′ overhangs (top) and DNA sequences of two potential joining events (bottom) are shown. (**F**) The ratio of repair junctions deriving from direct cohesive joining (dcj) of DNA ends to junctions deriving from blunting prior joining by filling up ssDNA overhangs (bpj) for HEK293 cells either expressing or non-expressing SAMHD1-Flag is shown. (**G**) DNA break structure of DNA plasmid substrate #2 carrying a 6 bp microhomology (top) and DNA sequences of two potential joining events (bottom) are shown. (**H**) The ratio of repair junctions deriving from direct joining (dj) of DNA ends to junctions deriving from MMEJ for HEK293 cells either expressing or non-expressing SAMHD1-Flag is shown. (mean values are indicated; significances were calculated by unpaired two-tailed t-tests from three independent experiments).

To further corroborate this finding, we analyzed the sequences of the individual intramolecular repair junctions of substrates #1 to #9 (Figure 2). By ranking the sequences according to abundance, we found that all repair junctions except substrate #5 (non-cohesive 5′ overhangs) shared the same predominant sequences, independent from presence or absence of SAMHD1-Flag (Figure 2A–D). However, in case of SAMHD1-Flag overexpression, we observed skewing towards a 1bp G:C microhomology within the repair junction of non-cohesive 5′ overhangs (substrate #5, Figure 2A–D). While in wt cells non-cohesive 5′ overhangs were preferentially blunted before joining, presence of overexpressed SAMHD1-Flag led to incomplete filling of the ssDNA overhangs prior joining, resulting in a 1bp shorter predominant repair junction, based on usage of a 1 nt microhomology.

For cohesive 5′ overhangs, we detected a preference for simple rejoining of the sticky ends. The second most frequent junction was generated by filling up the staggered ends prior joining of blunted DNA ends, resulting in a 4 nt longer repair junction. Nevertheless, we found that simple rejoining of cohesive 5′ overhangs was also more effective in SAMHD1 transfected cells (substrate #6, Figure 2A–D). This became apparent after analyzing the ratio of direct cohesive joining to sequences derived from joining of blunted ends (by filling up overhangs) prior joining (Figure 2E, F). However, we could not detect a general increase in MMEJ, as end structures carrying a 6 bp microhomology were not repaired at higher frequency in SAMHD1 transfected cells (substrate #2 in Figure 2G, H).

**Figure 3.**
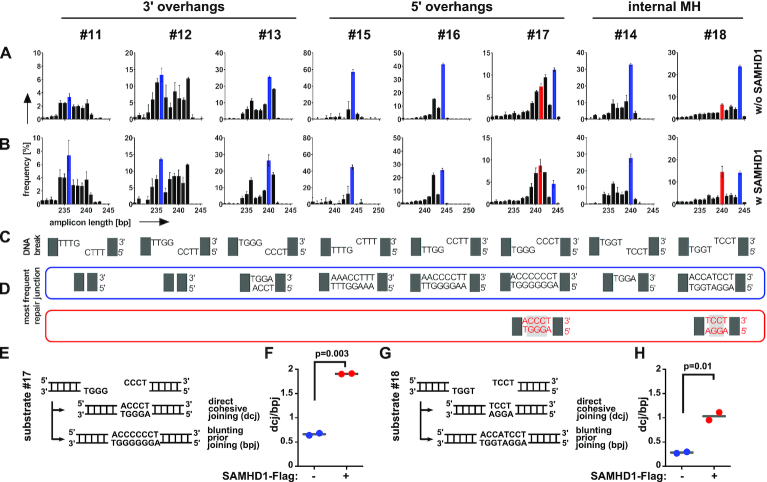
Repair of staggered ends with microhomologies by SAMHD1. (**A, B**) PCR-amplified repair junctions from SAMHD1-Flag non-expressing (A) or expressing (B) HEK293 cells were sequenced and the frequencies of the lengths of the respective sequences are shown for each DNA plasmid substrate (#11–#18). (**C**) The respective DNA break structures for DNA plasmid substrates are shown. (**D**) The sequences of the most frequent junctions for cells not expressing Flag-SAMHD1 are shown and framed in blue. The most frequent repair junctions for Flag-SAMHD1 expressing cells (if different to non-expressing cells), are shown below and framed in red (substrate #17 and #18). The color of the frame allocates the sequences to the respective bars shown in (A, B). (Bars are showing mean + SD from two independent experiments). (**E**) DNA break structure of DNA plasmid substrate #17 carrying cohesive 4 nt 5′ overhangs with 3bp microhomologies (top) and DNA sequences of two potential joining events (bottom) are shown. (**F**) The ratio of repair junctions deriving from direct cohesive joining (dcj) of DNA ends to junctions deriving from blunting prior joining (bpj) by filling up ssDNA overhangs (bpj) for HEK293 cells either expressing or non-expressing SAMHD1-Flag is shown. (**G**) DNA break structure of DNA plasmid substrate #18 carrying an internal 2bp microhomology (top) and DNA sequences of two potential joining events (bottom) are shown. (**H**) The ratio of repair junctions deriving from direct cohesive joining (dcj) of DNA ends to junctions deriving from blunting prior joining (bpj) for HEK293 cells either expressing or non-expressing SAMHD1-Flag is shown. (mean values are indicated; significances were calculated by unpaired two-tailed *t*-tests from two independent experiments).

To more thoroughly investigate the effect of SAMHD1 on repair of 3′ and 5′ overhangs, we constructed a second pool of linear plasmid substrates, carrying 1–3 complementary C:G nts at the end of non-cohesive 3′ and 5′ 4 nt overhangs (substrates #11–#13, #15–#17, Figure 3 and Supplementary Table S1). Here, we measured intramolecular repair in mock versus SAMHD1-Flag transfected HEK293 cells from two independent experiments as described above. By analyzing the sequences of the respective repair junctions, we again could not detect an impact of SAMHD1 overexpression on repair of 3′ overhangs (substrate #11–#13, Figure 3). In contrast, for repair of 5′ protruding ends, we confirmed a clear bias towards usage of microhomologies within the ssDNA overhangs, which was most pronounced for 3 nt microhomologies with the most frequent repair junction being 3 nt shorter in presence of SAMHD1-Flag (substrate #17, Figure 3). Corroboratively, repair junctions corresponding to partial or complete re-synthesis of cohesive overhangs from substrate #17 were markedly reduced in presence of Flag-SAMHD1 (Figure 3).

We next analyzed the impact of SAMHD1 on the repair of 4 nt 3′ and 5′ overhangs harboring an internal 2 nt CC:GG microhomology (substrate #14 and #18). Independent from SAMHD1 expression, microhomology was preferentially used for repair of 3′ overhangs with the 3′-terminal non-matching nt being resected and the gap refilled by the complementary nt (substrate #14, Figure 3). For 5′ overhangs, in absence of SAMHD1-Flag, the ssDNA region was preferentially refilled and converted to dsDNA followed by joining of blunted ends. However, when SAMHD1-Flag was present, the internal 2 nt microhomology was preferentially used for repair (substrate #18, Figure 3).

### End joining of Cas9 nickase induced chromosomal breaks is affected by physiologic SAMHD1 levels and requires catalytic activity

**Figure 4.**
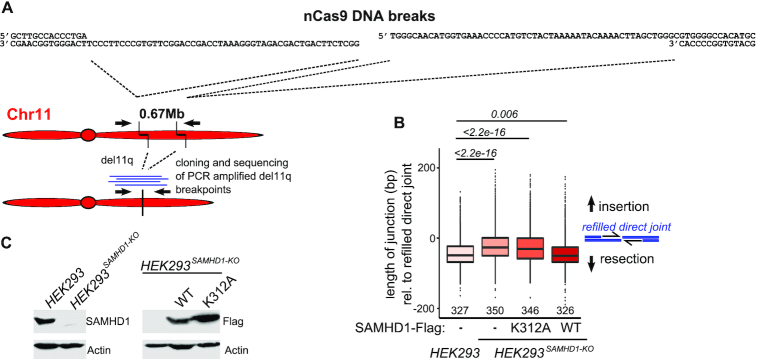
Induction of a chromosomal deletion at chr11q using nCas9. (**A**) Schematic representation of chr11 indicating nCas9 sites and primers for PCR amplification of breakpoint junctions. (**B**) Length of PCR-amplified amplicon-sequenced breakpoint junctions from HEK293 and HEK293*^SAMHD1-KO^* cells transfected with the indicated SAMHD1 variants (K312A or wt; *n* = 4 for all samples). A length corresponding to joining of blunted (filled up) 5′ overhangs is set to 0 bp. (**C**) Western blots of HEK293 and HEK293*^SAMHD1-KO^* cells (left), transiently transfected with the indicated constructs (right). For (B), median with interquartile range is shown in boxes, with whiskers extending the boxes with the largest/smallest value no further than 1.5 times of the interquartile range and other points plotted individually; significances were calculated by Mann-Whitney test and are indicated above the graph, medians are given above the x-axis.

Our previous experiments revealed that superphysiological levels of SAMHD1 affect EJ of artificial DNA substrates with 5′ overhangs. Next, we wanted to test whether physiological levels of SAMHD1 affect EJ of endogenous DNA breaks. Therefore, we generated a SAMHD1 knockout cell line in HEK293 cells (HEK293*^SAMHD1-KO^*). To monitor an effect on EJ of endogenous 5′ overhangs, we generated pairs of Cas9 nickases, which induce two 5′ staggered ends on chromosome 11q, which span a distance of 0.67 Mbp (Figure 4A). To analyze repair of these breaks by EJ, we characterized repair junctions descending from joining of the two distant DNA breaks, resulting in a chromosomal deletion (del11q). Therefore, we PCR-amplified breakpoint junctions from nCas9 induced del11q and analyzed individual junctions from amplicon sequencing approaches and from cloned PCR fragments by Sanger sequencing. First, we determined the mechanism by which nCas9 induced DNA breaks lead to del11q. We found that del11q was detected only when paired nicks on both sites (and thus two DSBs) were present, whereas a single nick combined with a paired nick was not sufficient for generating a chromosomal deletion (Supplementary Figure S2A, B). In addition, we tested the sensitivity of the generation of del11q towards NU7441, an inhibitor of DNA-PKcs, which is required for NHEJ. As shown in Supplementary Figure S2C, treatment of cells with NU7441 prior transfection with paired nCas9 constructs impeded induction of del11q, although nCas9 expression levels were not affected (Supplementary Figure S2C). In line with previous reports (24), these experiments pointed to a NHEJ-dependent mechanism as the major pathway contributing to nCas9 induced del11q. To examine the impact of SAMHD1 on del11q, we performed amplicon-sequencing of PCR-amplified del11q junctions from HEK293 versus HEK293*^SAMHD1-KO^* cells and HEK293*^SAMHD1-KO^* cells transfected with wt SAMHD1 or catalytically dead K312A mutant, lacking dNTPase and nuclease activity (8). We found that in presence of endogenous SAMHD1, junctions were significantly shorter than in the knockout cells or in cells expressing the catalytically dead K312A variant (Figure 4B). In line with our plasmid based repair assays, we observed that overexpression of SAMHD1 induced an even more pronounced skewing towards shorter repair junctions compared to physiological levels, showing that SAMHD1 expression levels predict its effect on EJ (Figure 4B). To validate results from amplicon-sequencing, we cloned individual PCR-amplified del11q junctions and analyzed them by Sanger sequencing. Thereby, we could confirm the results from amplicon-sequencing and found that junctions from knockout cells or K312A expressing cells were significantly longer than wt-expressing or wt-overexpressing samples (Supplementary Figure S3, Supplementary table S2). Furthermore, we analyzed microhomologies at cloned unique del11q junctions, which revealed a typical NHEJ-dependent pattern with predominant 0–1 bp microhomologies independent of SAMHD1 expression (Supplementary Figure S3B and Supplementary Table S2). Western blotting confirmed similar expression levels between wt and K312A mutant of SAMHD1 from transfected samples (Figure 4C). This shows that catalytic activity is important for the observed effect on EJ. Strikingly, by analyzing cloned, Sanger-sequenced del11q junctions we found many duplications and insertions at breakpoint junctions from SAMHD1 deficient or K312A expressing cells. The duplications derived from resynthesized DNA overhangs at nCas9 target sites, whereas the insertions mapped either to distant genomic regions from other chromosomes or to transfected plasmid DNA (SAMHD1 or nCas9 expressing plasmids; Supplementary Figure S3C; Supplementary Table S2). Some junctions featured complex arrangements of repetitive duplications and insertions, which were not observed in junctions from HEK293 cells or SAMHD1 deficient cells complemented with wt SAMHD1 expressing plasmids (Supplementary Figure S4; Supplementary Table S2).

### Increase of the cellular dNTP pool enhances insertions at nickase induced chromosomal junctions

**Figure 5.**
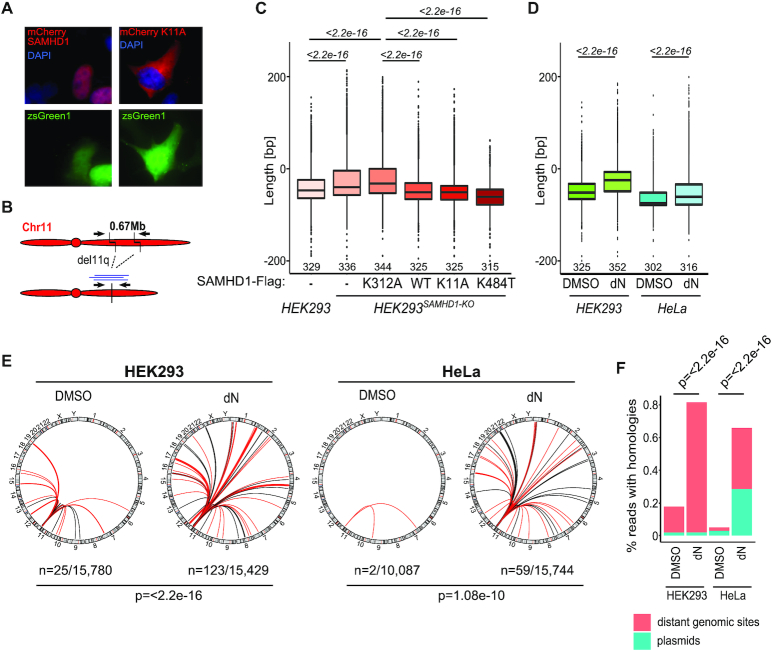
Elevated dNTP pool increases insertions at repair junctions. (**A**) HEK293*^SAMHD1-KO^* cells were transiently transfected with constructs encoding SAMHD1 and SAMHD1 K11A mutants N-terminally fused to mCherry together with del11q inducing nCas9 constructs encoding zsGreen1 as separately transcribed gene. (**B**) Schematic representation of nCas9 induced del11q and primers for PCR amplification of breakpoint junctions. (**C**) Length of PCR-amplified amplicon-sequenced breakpoint junctions from HEK293 and HEK293*^SAMHD1-KO^* cells transfected with the indicated SAMHD1 variants (K11A, K484T, K312A or wt) (*n* = 4 for all samples, except K484T *n* = 3) and (**D**) from HEK293 and HeLa cells grown in excessive dN to increase intracellular dNTP pools (*n* = 2 for HEK293 cells and *n* = 3 for HeLa cells). Median with interquartile range is shown in boxes, with whiskers extending the boxes with the largest/smallest value no further than 1.5 times of the interquartile range and other points plotted individually; significances were calculated by Mann–Whitney test and are indicated above the graph, medians are given above the x-axis. (**E**) Circos plots were generated from data in Figure 5D, which show the genome as circle with ribbons indicating the homologies of inserted DNA at the del11q repair junction to the respective distant genomic regions. Ribbons in red indicate that the insertions map to the genome ambiguously, with only one randomly chosen homology shown (detailed mapping results are summarized in Supplementary table S4; *n* = number of junctions with distant homologies/total number of unique reads analyzed). (**F**) Bars show the percentage of all amplicon-sequencing reads with insertions mapping to distant genomic sites or to transfected plasmids. Significances in (E, F) were calculated by Fisher's exact test.

To further investigate whether the effect of SAMHD1 on EJ is solely depending on its catalytic dNTPase activity and independent from its scaffold function at DNA ends, we repeated our nCas9 experiment including two catalytically active SAMHD1 mutants: one that fails to recruit the CtIP endonuclease to DNA breaks due to a K484T mutation (14) and another that is excluded from the nucleus due to a K11A mutation within the nuclear localization sequence (NLS) (25,26). In addition, we performed this nCas9 assay also in wildtype HEK293 cells and a second cell line (HeLa cells) in presence of high amounts of deoxynucleosides (dNs) as metabolic dNTP precursors to artificially increase the intracellular dNTP pool, mimicking high dNTP levels upon SAMHD1 deficiency (27). In line with previous reports, a SAMHD1 K11A mutant fused to mCherry showed primarily cytoplasmic localization, also in presence of nCas9 endocucleases (Figure 5A). Next, we PCR amplified breakpoint junctions from nCas9 induced del11q (schematically depicted in Figure 5B) in HEK293*^SAMHD1-KO^* cells either expressing wt SAMHD1 or mutants K11A, K484T or K312A and in HEK293 cells as well as HeLa cells supplemented with dNs to increase dNTP levels. PCR amplified junctions were amplicon-sequenced on a MiSeq next generation sequencing platform and amplicon lengths were calculated. Again, absence of SAMHD1 as well as presence of a catalytically dead K312A variant resulted in significantly longer repair junctions, whereas expression of the cytosolic K11A mutant and mutant K484T induced shorter junctions similar to what we observed in wt SAMHD1 expressing cells (Figure 5C). Again, expression of low levels of endogenous SAMHD1 in wt HEK293 cells resulted in intermediate junction lengths (Figure 5C). These results were confirmed by analyzing Sanger-sequences from cloned PCR-amplified del11q junctions (Supplementary Figure S5; Supplementary table S3). Corroboratively, increasing dNTP levels by dN addition in two cell lines (HEK293 and HeLa) also yielded significantly longer junctions, similar to loss of SAMHD1 (Figure 5D). Increased intracellular dNTPs in SAMHD1 knockout HEK293 cells as well as upon dN supplementation in HEK293 and HeLa cells were confirmed by an EvaGreen-based detection assay (Supplementary Figure S6) (17). Notably, dN addition in SAMHD1 knockout HEK293 cells did further increase dNTP levels (Supplementary Figure S6) but did not lead to a further increase in the length of amplified breakpoint junctions, showing that the observed effect on insertions had reached a plateau (Supplementary Figure S7). Finally, we wanted to examine all sequences for presence of insertions that mapped to distant genomic regions or to transfected plasmids. To systematically detect insertions mapping to distant DNA sites at del11q junctions from our amplicon-sequencing dataset, we developed automated blast searches on all unique sequences longer than 376nt, corresponding to repair joints descending from refilled overhangs. Thereby, we found a significantly higher percentage of junctions with insertions mapping to distant sites in samples lacking SAMHD1 or expressing K312A variant compared to SAMHD1 proficient cells or cells expressing the catalytically active SAMHD1 K11A or K484T mutant (Supplementary Figure S8; Supplementary table S4). Corroboratively, increased dNTP levels upon dN addition in HEK293 and HeLa cells also yielded a higher percentage of junctions with insertions mapping to distant genomic regions or to transfected plasmids (Supplementary Figure S5E and F; Supplementary table S4). Strikingly, many of these genomic regions were previously defined as common fragile sites, which are sensitive to replication stress and vulnerable for DNA breakage, with some of these sites mapping to cancer related genes (Supplementary Table S5) (22).

## DISCUSSION

SAMHD1 is a remarkable enzyme with an increasing number of attributed biologically important tasks. Originally detected as dNTPase, SAMHD1 was implicated in HIV-restriction by minimizing the dNTP pool to a level incompatible with reverse transcription of the viral genome. As even small variations in the level of dNTP concentrations can affect precision and accuracy of DNA polymerase activity, a further role for SAMHD1 in replication fidelity and cell cycle regulation was suggested (28–31). Based on its dNTPase activity, SAMHD1 was also reported to detoxify DNA base analogs, currently used in cancer treatment, thus, serving as biomarker predicting low response to these drugs (32).

In addition to this function, SAMHD1 was recently reported to be recruited to stalled replication forks, degrading nascent DNA by attracting Mre11 exonuclease, which enables efficient replication restart and avoids release of ssDNA to the cytosol, which otherwise would induce a robust interferon (IFN)-response (33). Germline mutations in the *SAMHD1* gene have been associated with the Aicardi-Goutières syndrome (AGS), a hereditary autoimmune encephalopathy, suggesting that constant immune-stimulating IFN signaling in absence of SAMHD1 is the underlying basis for aberrant inflammation in AGS (33,34). Furthermore, SAMHD1 is an important factor for homologous DNA repair, as it binds to DSBs and recruits the endonuclease CtIP, which generates 3′ ssDNA overhangs for strand invasion during homologous recombination. Notably, SAMHD1 executes its role in homologous DNA repair independent from its dNTPase activity (14). Many of these functions, particularly its role in homologous repair and in regulating the dNTP pool are important for genome integrity, suggesting an important role in cancer development. Indeed, downregulation or recurrent SAMHD1 mutations were found in some cancer entities like chronic lymphocytic leukemia, colon cancer and T-cell prolymphocytic leukemia and were particular increased in chemorefractory cases, which classifies SAMHD1 as cancer gene (13,30,35,36). Our own data show for the first time a previously undefined role for SAMHD1 in DNA end joining of DSBs. We provide evidence that in presence of catalytically active SAMHD1, breakpoint junctions are shorter, featuring less DNA resynthesis. This effect was restricted to DNA ends with 5′ overhangs, as only these structures provide 3′ hydroxyl-ends for polymerase-dependent gap filling. This effect was more pronounced in presence of overexpressed SAMHD1 and depended on its catalytic activity, suggesting that the levels of dNTPs regulated by SAMHD1 dictate the velocity of DNA end resynthesis by DNA-polymerases. This assumption is corroborated by our finding that increasing the pool of dNTPs by dN supplementation enhances the extent of nucleotide insertions at breakpoint junctions similar to SAMHD1 deficiency. Furthermore, a SAMHD1 K11A mutant, that is excluded from the nucleus while retaining catalytic functions (25,26), as well as a K484T mutant that fails to interact with CtIP (14) are also leading to short repair junctions, similar to those observed in wt SAMHD1 expressing cells, which suggests that solely intracellular dNTP levels regulate the extent of insertions at EJ dependent repair junctions. In line with this, elevated dNTP levels were reported to increase DNA replication rates and polymerase activities, particularly in presence of DNA lesions (28,37,38).

Most strikingly, many of the insertions observed upon SAMHD1 loss were homologous to distant genomic sites or to transfected plasmids. Insertion of nuclear or mitochondrial DNA into breakpoints from chromosomal rearrangements is commonly observed in cancer cells and constitutes a threat to genome integrity (39–41). Even in normal cells, insertions of mitochondrial DNA into genomic regions were discerned, pointing to an important mechanism that shapes the evolution of genomes (42). Insertions can either emerge from direct incorporation of DNA fragments from distant sites, or by copying distant DNA (fragments) without causing a deletion at the originating position, which is therefore called templated insertion. According to the current view, templated insertions originate from DNA released from damaged genomic or episomal/mitochondrial sites or otherwise, it may derive from RNA transcripts, which were reversely transcribed by LINE-1 (long interspersed element-1) endonuclease and reverse transcriptase (ORF2) (41,43). In either case, the DNA fragments can be directly inserted at the repair joint or can serve as template for DNA polymerases at DSB sites. Corroboratively, many insertions in our analysis mapped to fragile sites, which are chromosomal regions susceptible to DNA breakage (22). As yeast experiments revealed that Pol4 (a pol X family polymerase) was responsible for insertion of DNA fragments (41), presumably one of the pol X family polymerases, comprising pol β, pol λ, pol μ and terminal deoxynucleotidyl transferase (TdT) are mediating this synthesis in human cells (44). As our study reveals that insertions were increased in absence of catalytically active SAMHD1 or in presence of elevated dNTP pools, we propose that super-physiological dNTP levels may increase DNA polymerase resynthesis rates leading to increased refilling of staggered DNA ends and synthesis over inserted DNA templates prior end ligation. Strikingly, our data are in line with a parallel report, addressing SAMHD1 in class switch recombination of antibody constant regions in B cells, which was published during finalization of our manuscript (15). In this parallel work, it was concomitantly shown that absence of SAMHD1 and high intracellular dNTP levels lead to aberrant insertions at EJ dependent switch junctions.

Summarizing, our data further support a previously undefined role for SAMHD1 in preserving genome integrity and draw light on how loss of SAMHD1 and aberrant dNTP levels could contribute to cancer formation, clonal cancer evolution and treatment resistance promoted by aberrant insertions at repair joints.

## DATA AVAILABILITY

All sequencing data, which have been deposited in the ArrayExpress database at EMBL-EBI (www.ebi.ac.uk/arrayexpress) under accession number E-MTAB-8382.

## Supplementary Material

gkab051_Supplemental_Files
