# Supplementary material for: SAMHD1 restrains aberrant nucleotide insertions at repair junctions generated by DNA end joining
Source: Nucleic Acids Res. 2021 Feb 16;49(5):2598–608. doi: 10.1093/nar/gkab051 (PMC7969033; doi:10.1093/nar/gkab051)
Supplement: gkab051_Supplemental_Files [file gkab051_supplemental_files.zip › Supplementary Figures.pdf]

Supplementary Figures and Table legends

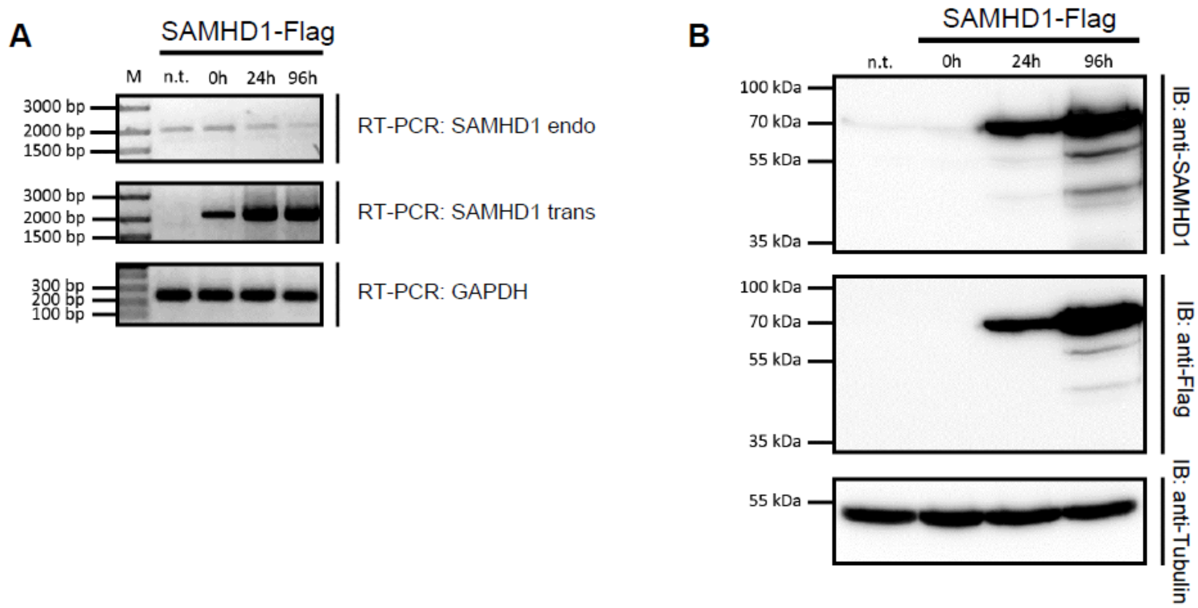

Figure S1. Expression of SAMHD1 in 293 cells. (A) RT-PCR of SAMHD1-flag transfected and untransfected (n.t.) HEK293 cells at indicated time points. **(B)** Western blot using SAMHD1-specific and anti-Flag antibodies of SAMHD1-flag transfected and untransfected (n.t.) HEK293 cells at indicated time points.

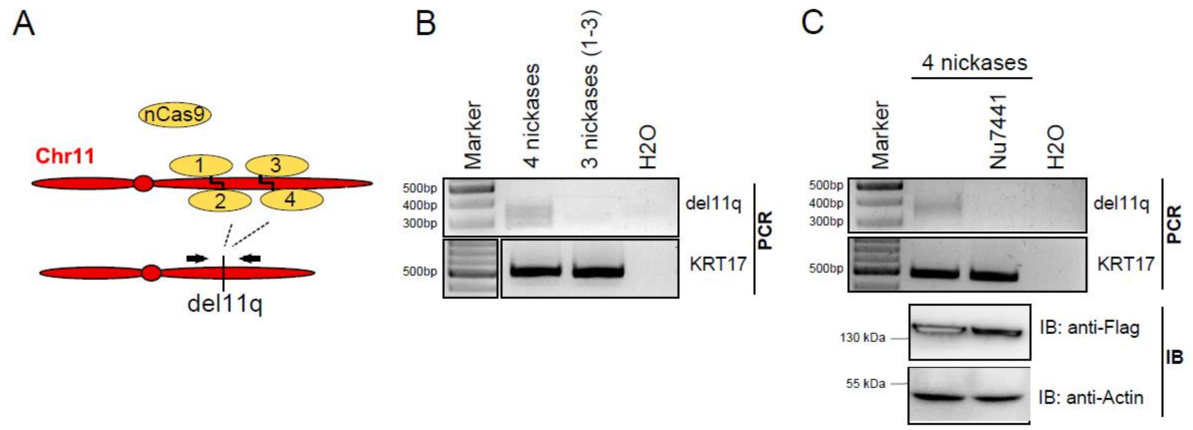

Figure S2. nCas9 induced del11q is mediated by joining of DNA-DSBs. (A) Schematic representation of del11q induced by two pairs of nCas9 nickases (1-4). **(B)** Two pairs of nicks are necessary to induce del11q. HEK293 cells were either transfected with all 4 or only with 3 nickases and presence of del11q was determined with PCR (del11q). A control PCR on KRT17 is shown on the lower panel. **(C)** Induction of del11q is inhibited by NU7441. HEK293 cells were transfected with nickases in presence or absence of DNA-PKc inhibitor NU7441 and del11q was determined by PCR on 60ng input DNA. A control PCR on KRT17 is shown on the lower panel. As a further control, western blotting on Flag-tagged nCas9 was performed from cell lysates.

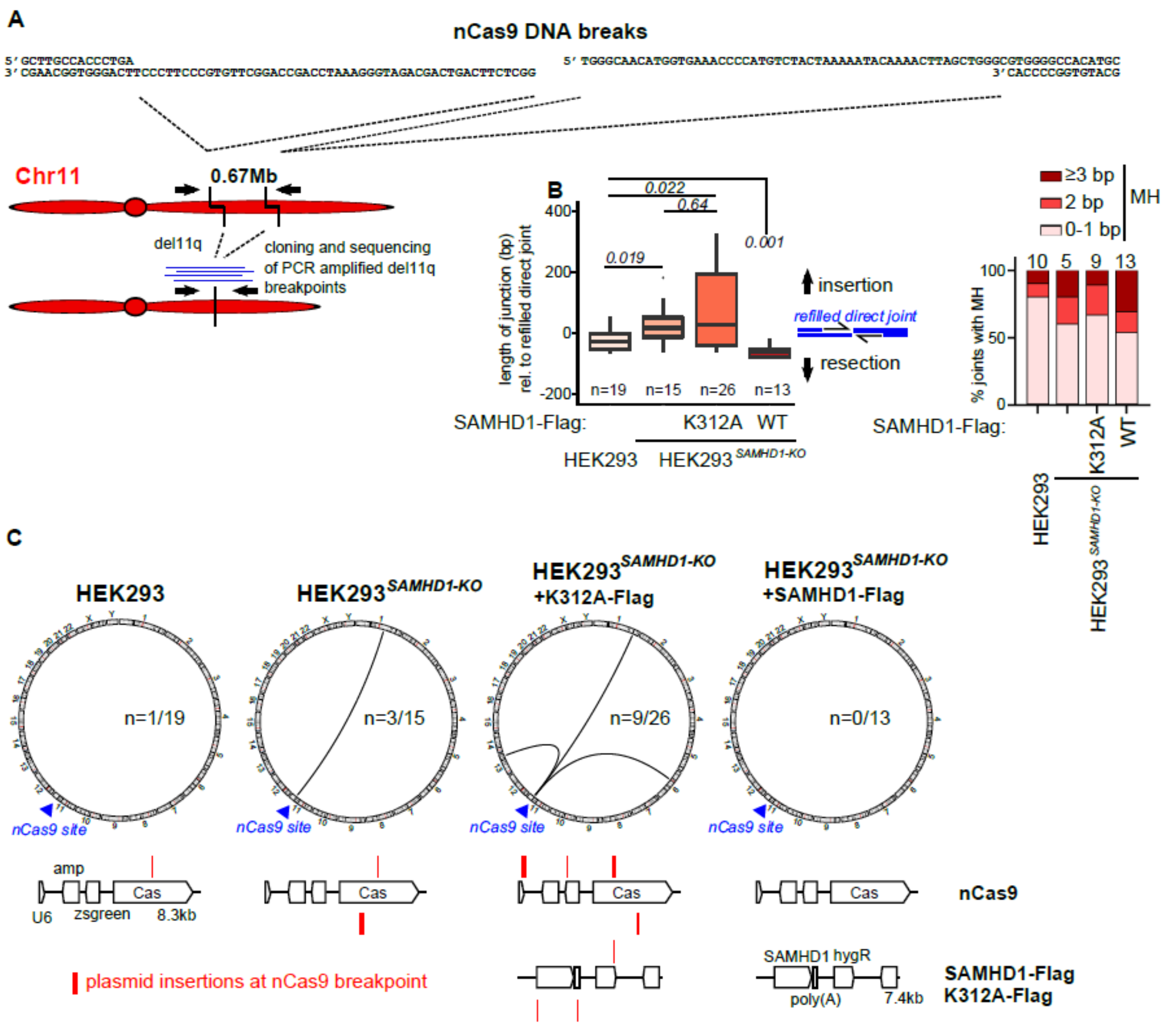

Figure S3. Induction of a chromosomal deletion at chr11q using nCas9. (A) Schematic representation of chr11 indicating nCas9 sites and primers for PCR amplification of breakpoint junctions. **(B)** Length of Topo-cloned PCR-amplified breakpoint junctions from HEK293 and HEK293*SAMHD1-KO* cells transfected with the indicated SAMHD1 variants (K312A or wt). A length corresponding to joining of blunted (filled up) 5' overhangs is set to 0bp. Only unique sequences corresponding to joints are shown (n= number of sequences analyzed). Right panel: graph shows the length of microhomologies at breakpoint junctions from Sanger sequences provided as supporting Table S2. Junctions with insertions were excluded from the analysis. **(C)** DNA insertions at breakpoint junctions map to distant chromosomal regions or to transfected plasmids. Circos plots show the genome as circle with ribbons indicating the homologies of inserted DNA at the del11q repair junction to the respective distant genomic regions (n= number of junctions with distant homologies/total number of junctions analyzed). Inserted DNA deriving from transfected plasmids are indicated as red rectangles above (+ orientation) or below (- orientation) the schematically depicted plasmids (not to scale). In (B), median with interquartile range is shown in boxes, with whiskers extending the boxes with the largest/smallest value no further than 1.5 times of the interquartile range and other points plotted individually; significances were calculated by Mann-Whitney test.

GGCCGGGGGGAGCTTGCCACCCTGAAGGGAAGGGCACAAGCCTGGCTGGATTTCCCATCTGCCCCCAT
GTCTACTAAAAATACAAAACTTAAGGGAAGGGCACAAGCCTGGCTGGATTTCCCATCTGCCCCCATGT
CTACTAAAAATACAAAACTTAGCTGGCGGAATCGGCTCGCTGCATGGTACCAAGCTTGGTGGCGGCCT
AGAATAGAATGACACCTACTCAGACAATGCGATGCAATTTCCTCATTTTATTAGGCGACGCAATCGTC
CGATCCGGAGCCGGGACTGTCGGGCGTACACAAATCGCCCGCAGGGAAGGGCACAAGCCTGGCTGGAT
TTCCCATCTGCCCCCATGTCTACTAAAAATACAAAACTTAAGGGAAGGGCACAAGCCTGGCTGGATTT
CCCATCTGCCCCCATGTCTACTAAAAATACAAAACTTAGCTGGGCGTGGGGCCACATGCTTTTGTAAT

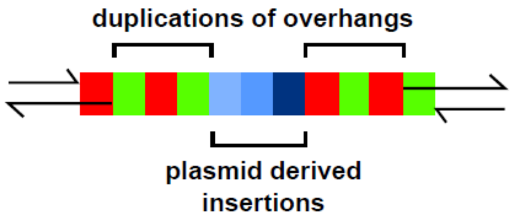

Figure S4. Complex insertions at nCas9 induced breakpoints from K312A expressing HEK293 cells. Sequences of duplications from overhangs and inserted plasmid DNA from a breakpoint junction deriving from K312A expressing HEK293 cells are indicated.

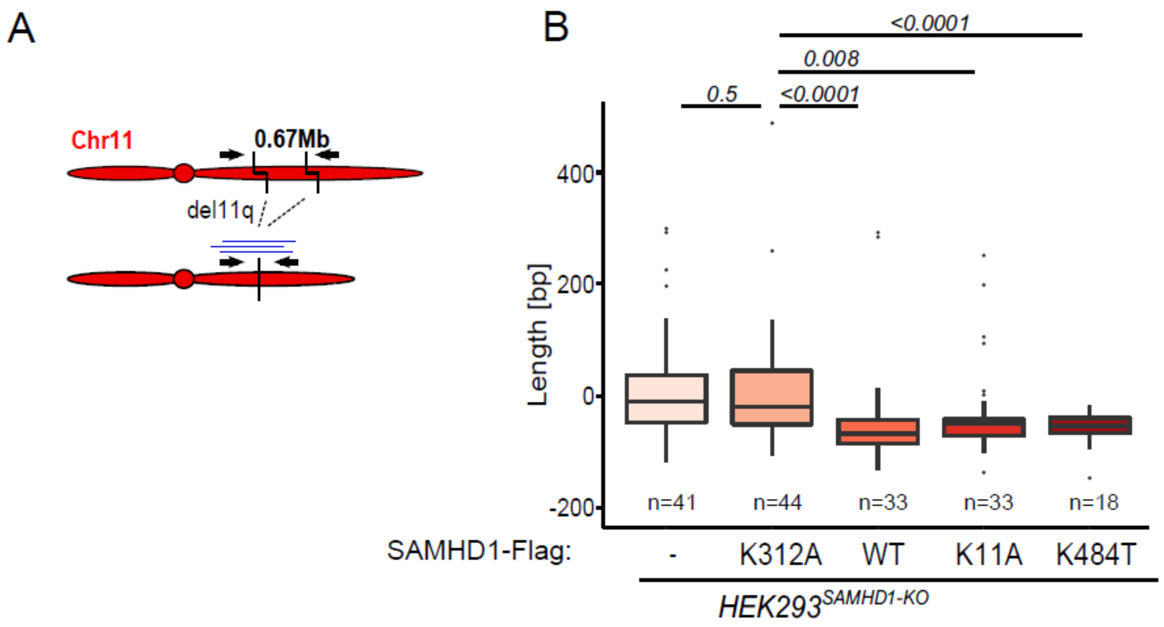

Figure S5. Induction of del11q by nCas9 in presence of SAMHD1 mutants. (A) Schematic representation of chr11 indicating nCas9 sites and primers for PCR amplification of breakpoint junctions. **(B)** Length of TOPO-cloned PCR-amplified breakpoint junctions from HEK293$^{SAMHD1\text{-}KO}$ cells transfected with the indicated SAMHD1 variants (from 4 independent experiments). Gel purified PCR products were TOPO-cloned and individual colonies were Sanger-sequenced (n= number of unique sequences analyzed). A length corresponding to joining of blunted (filled up) 5’ overhangs is set to 0bp. Median with interquartile range is shown in boxes, with whiskers extending the boxes with the largest/smallest value no further than 1.5 times of the interquartile range and other points plotted individually; significances were calculated by Mann-Whitney test.

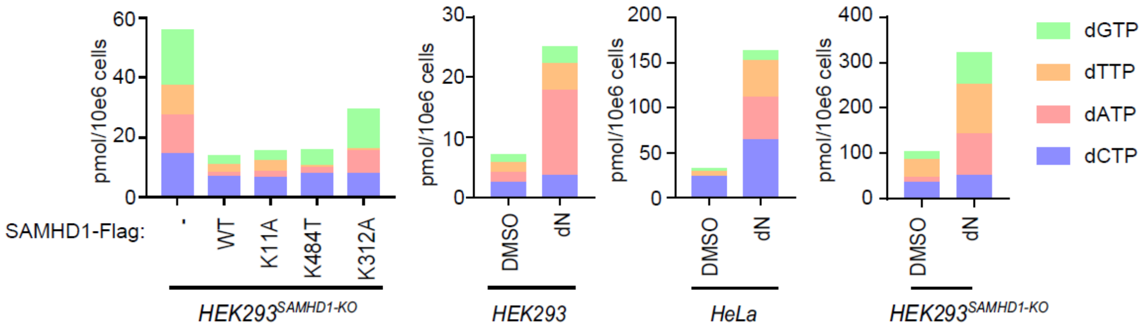

Figure S6. Measurement of cellular dNTP levels. Intracellular dNTP levels extracted from the respective samples (knockout HEK293 cells transfected with the respective SAMHD1 constructs or wt HEK293 and HeLa cells supplemented with dN) were quantified by an EvaGreen based detection assay. Results from one of two experiments are shown.

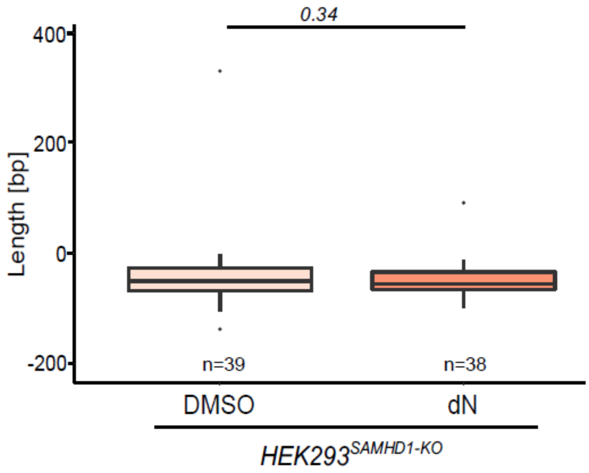

Figure S7. Analysis of del11q in HEK293 SAMHD1 knockout cells supplemented with dN. Length of PCR-amplified breakpoint junctions from HEK293 SAMHD1 knockout cells grown in excessive dN to increase intracellular dNTP pools (from 2 independent experiments). Gel purified PCR products were TOPO-cloned and individual colonies were Sanger-sequenced (n= number of unique sequences analyzed). A length corresponding to joining of blunted (filled up) 5’ overhangs is set to 0bp. Median with interquartile range is shown in boxes, with whiskers extending the boxes with the largest/smallest value no further than 1.5 times of the interquartile range and other points plotted individually; significances were calculated by Mann-Whitney test.

A

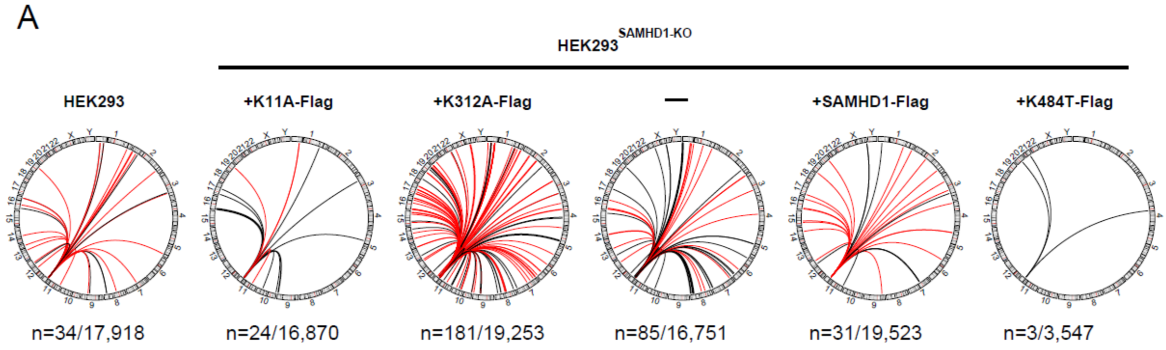

	KO	KO + SAMHD1	KO + K11A	KO + K312A	KO + K484T	HEK293
KO		3.49E-09	1.9E-09	1.73E-06	1.04E-04	4.46E-07
KO + SAMHD1			0.787	<2.2E-16	0.4719	0.0035
KO + K11A				<2.2E-16	0.6094	0.2953
KO + K312A					2.88E-10	<2.2E-16
KO + K484T						0.2639

B

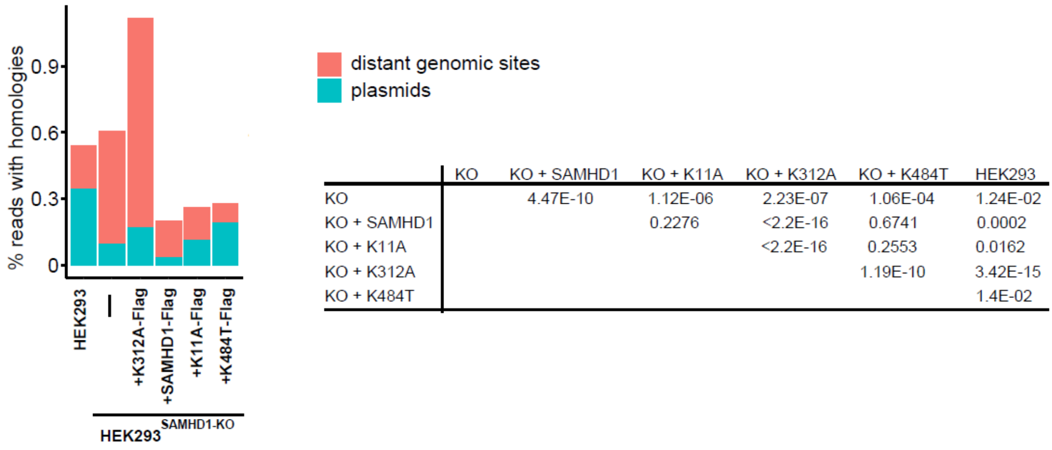

	KO	KO + SAMHD1	KO + K11A	KO + K312A	KO + K484T	HEK293
KO		4.47E-10	1.12E-06	2.23E-07	1.06E-04	1.24E-02
KO + SAMHD1			0.2276	<2.2E-16	0.6741	0.0002
KO + K11A				<2.2E-16	0.2553	0.0162
KO + K312A					1.19E-10	3.42E-15
KO + K484T						1.4E-02

Figure S8. Elevated dNTP pool increases insertions at repair junctions. (A) Circos plots were generated from PCR-amplified amplicon-sequenced repair joints from HEK293$^{SAMHD1\text{-}KO}$ cells transfected with the indicated Flag-SAMHD1 constructs and nCas9 constructs (repair joint lengths depicted in Fig 5C), which show the genome as circle with ribbons indicating the homologies of inserted DNA at the del11q repair junction to the respective distant genomic regions. Ribbons in red indicate that the insertions map to the genome ambiguously, with only one randomly chosen homology shown (detailed mapping results are summarized in table S4; n= number of junctions with distant homologies/total number of reads analyzed). Data were gained from pooled amplicon sequencing approaches from 4 independent experiments, except K484T n=3. **(B)** Bars show the percentage of all amplicon-sequencing reads with insertions mapping to distant genomic sites or to transfected plasmids. Significances were calculated by Fisher's exact test.

Supplementary Table legends

Table S1. List of plasmid inserts used for the plasmid based repair assay.

Table S2. Sanger sequences from supporting Figure S3.

Table S3. Sanger sequences from supporting Figure S5.

Table S4. List of reads (show in Fig 5 and Fig S8) with insertions mapping to indicated distant genomic sites or transfected plasmids.

Table S5. List of reads (from table S4) with insertions mapping to fragile sites. In case insertions map to multiple homologies within the genome or to plasmids, only homologies depicted in circos plots (Fig 5 and Fig S8), which were selected randomly beforehand (see Methods), are depicted in the table.
