# Supplementary material for: SAMHD1 restrains aberrant nucleotide insertions at repair junctions generated by DNA end joining
Source: Nucleic Acids Res. 2021 Feb 16;49(5):2598–608. doi: 10.1093/nar/gkab051 (PMC7969033; doi:10.1093/nar/gkab051)
Supplement: gkab051_Supplemental_Files [file gkab051_supplemental_files.zip › Table S1.docx]

Supplementary Table S1

List of plasmid inserts used for the plasmid based repair assay.

Restriction enzymes for linearization of plasmids are indicated and respective restriction sites are italicized within the sequence.

| 1 | EcoRV/SmaI | GCAAATGGCATTCTGACATCCAAAATGGAGAAAAAAATCACTGGATATACCACCGTTGATATATCCCAATGGCATCGTAAAGAACATTTTGAGGCATTTCAGTCAGTTGCTCAATGTACCTATAACCAGACCGTTCAGCTgatatcCGGCCTTTTTAAAGACCGTAAAGAAAAATAAGCACAAGTTTTATCCGGCCTTTATTCACATTCTTGCCCGCCTGATGAATGCTCATCCGGAgTTCCGTATGGCAATGcccgggAAAGACGGTGAGCTGGTGATATGGGATAGTGTTCACCCTTGTTACACCGTTTTCCATGAGCAAACTGAAACGTTTTCATCGCTCTGGAGTGAATACCACGACGATTTCCGGCAGTTTCTGCTTGTCAATTGGAACCAGTC |
| --- | --- | --- |
| 2 | EcoRV/AfeI | GCAAATGGCATTCTGACATCCATTCGCAAGATGTGGCGTGTTACGGTGAAAACCTGGCCTATTTCCCTAAAGGGTTTATTGAGAATATGTTTTTCGTCTCAGCCAATCCCTGGGTGAGTTTCACCAGTTTTGATTTAAACgctgatatcCGGCCTTTTTAAAGACCGTAAAGAAAAATAAGCACAAGTTTTATCCGGCCTTTATTCACATTCTTGCCCGCCTGATGAATGCTCATCCGGAgTTCCGTATGGCAATGagcgctgatTATGGACAACTTCTTCGCCCCCGTTTTCACCATGGGCAAATATTATACGCAAGGCGACAAGGTGCTGATGCCGCTGGCGATTCAGGTTCATCATGCCGTCTGTGATGGCTTCCATGTCGGCTTGTCAATTGGAACCAGTC |
| 3 | PstI/BstXI | GCAAATGGCATTCTGACATCCTGATATGTCTAGATTAGATAAAAGTAAAGTGATTAACAGCGCATTAGAGCTGCTTAATGAGGTCGGAATCGAAGGTTTAACAACCCGTAAACTCGCCCAGAAGCTAGGTGTAGAGCAGCctgcagCGGCCTTTTTAAAGACCGTAAAGAAAAATAAGCACAAGTTTTATCCGGCCTTTATTCACATTCTTGCCCGCCTGATGAATGCTCATCCGGAgTTCCGTATGGCAATGCCAgacgtcTGGCTACATTGTATTGGCATGTAAAAAATAAGCGGGCTTTGCTCGACGCCTTAGCCATTGAGATGTTAGATAGGCACCATACTCACTTTTGCCCTTTAGAAGGGGAAAGCTGGCAAGATTTTGCTTGTCAATTGGAACCAGTC |
| 4 | BstXI | GCAAATGGCATTCTGACATCCTTACGTAATAACGCTAAAAGTTTTAGATGTGCTTTACTAAGTCATCGCGATGGAGCAAAAGTACATTTAGGTACACGGCCTACAGAAAAACAGTATGAAACTCTCGAAAATCAATTAGCCCAtagcttTGGCGGCCTTTTTAAAGACCGTAAAGAAAAATAAGCACAAGTTTTATCCGGCCTTTATTCACATTCTTGCCCGCCTGATGAATGCTCATCCGGAgTTCCGTATGGCAATGCCAtagcttTGGCTTTTTATGCCAACAAGGTTTTTCACTAGAGAATGCATTATATGCACTCAGCGCTGTGGGGCATTTTACTTTAGGTTGCGTATTGGAAGATCAAGAGCATCAAGTCGCTAAAGAAGAAAGCTTGTCAATTGGAACCAGTC |
| 5 | SpeI/BglII | GCAAATGGCATTCTGACATCCGGGAAACACCTACTACTGATAGTATGCCGCCATTATTACGACAAGCTATCGAATTATTTGATCACCAAGGTGCAGAGCCAGCCTTCTTATTCGGCCTTGAATTGATCATATGCGGATTAactagtCGGCCTTTTTAAAGACCGTAAAGAAAAATAAGCACAAGTTTTATCCGGCCTTTATTCACATTCTTGCCCGCCTGATGAATGCTCATCCGGAgTTCCGTATGGCAATGagatctCATGGCCAAGTTGACCAGTGCCGTTCCGGTGCTCACCGCGCGCGACGTCGCCGGAGCGGTCGAGTTCTGGACCGACCGGCTCGGGTTCTCCCGGGACTTCGTGGAGGACGACTTCGCCGGCTTGTCAATTGGAACCAGTC |
| 6 | HindIII | GCAAATGGCATTCTGACATCCGTGTGGTCCGGGACGACGTGACCCTGTTCATCAGCGCGGTCCAGGACCAGGTGGTGCCGGACAACACCCTGGCCTGGGTGTGGGTGCGCGGCCTGGACGAGCTGTACGCCGAGTGGTCGaagcttCGGCCTTTTTAAAGACCGTAAAGAAAAATAAGCACAAGTTTTATCCGGCCTTTATTCACATTCTTGCCCGCCTGATGAATGCTCATCCGGAgTTCCGTATGGCAATGaagcttGAGGTCGTGTCCACGAACTTCCGGGACGCCTCCGGGCCGGCCATGACCGAGATCGGCGAGCAGCCGTGGGGGCGGGAGTTCGCCCTGCGCGACCCGGCCGGCAACTGCGTGCACTTCGTGCTTGTCAATTGGAACCAGTC |
| 7 | BstXI/NheI | GCAAATGGCATTCTGACATCCTGGTGAGCGGCCTGCTGAAGGAGAGTATGCGCATCAAGATGTACATGGAGGGCACCGTGAACGGCCACTACTTCAAGTGCGAGGGCGAGGGCGACGGCAACCCCTTCGCCGGCACCCAGCCAtaacttTGGCGGCCTTTTTAAAGACCGTAAAGAAAAATAAGCACAAGTTTTATCCGGCCTTTATTCACATTCTTGCCCGCCTGATGAATGCTCATCCGGAgTTCCGTATGGCAATGgctagcAGCATGAGAATCCACGTGACCGAGGGCGCCCCCCTGCCCTTCGCCTTCGACATCCTGGCCCCCTGCTGCGAGTACGGCAGCAGGACCTTCGTGCACCACACCGCCGAGATCCCCGACTTGCTTGTCAATTGGAACCAGTC |
| 8 | BstXI/EcoRV | GCAAATGGCATTCTGACATCCCTTCAAGCAGAGCTTCCCCGAGGGCTTCACCTGGGAGAGAACCACCACCTACGAGGACGGCGGCATCCTGACCGCCCACCAGGACACCAGCCTGGAGGGCAACTGCCTGATCTACAAGGCCAattccgTGGCGGCCTTTTTAAAGACCGTAAAGAAAAATAAGCACAAGTTTTATCCGGCCTTTATTCACATTCTTGCCCGCCTGATGAATGCTCATCCGGAgTTCCGTATGGCAATGgatatcTGAAGGTGCACGGCACCAACTTCCCCGCCGACGGCCCCGTGATGAAGAACAAGAGCGGCGGCTGGGAGCCCAGCACCGAGGTGGTGTACCCCGAGAACGGCGTGCTGTGCGGCCGGAACGCTTGTCAATTGGAACCAGTC |
| 9 | NcoI/EcoRV | GCAAATGGCATTCTGACATCCGTGATGGCCCTGAAGGTGGGCGACCGGCACCTGATCTGCCACCACTACACCAGCTACCGGAGCAAGAAGGCCGTGCGCGCCCTGACCATGCCCGGCTTCCACTTCACCGACATCCGGCTccatggCGGCCTTTTTAAAGACCGTAAAGAAAAATAAGCACAAGTTTTATCCGGCCTTTATTCACATTCTTGCCCGCCTGATGAATGCTCATCCGGAgTTCCGTATGGCAATGgatatcAACCAGTAACGTTATACGATGTCGCAGAGTATGCCGGTGTCTCTTATCAGACCGTTTCCCGCGTGGTGAACCAGGCCAGCCACGTTTCTGCGAAAACGCGGGAAAAAGTGGAAGCGGCGGCTTGTCAATTGGAACCAGTC |
|  |  |  |
| 11 | BstXI | GCAAATGGCATTCTGACATCCCTGCCACGCAGCCGGAGGAGCACAGGGGATCGGAGGAGGCGTCGACGATCCGCCGGCTGAAGGAGGAAAACGACGGACTAAAGCAGCGCGTGATGGCCTTGCAGCAACAGCACGACCTCCCCAttttgtTGGCGGCCTTTTTAAAGACCGTAAAGAAAAATAAGCACAAGTTTTATCCGGCCTTTATTCACATTCTTGCCCGCCTGATGAATGCTCATCCGGAgTTCCGTATGGCAATGCCAagaaaaTGGAGCAGGACGCGATGGCCAAGGTTCCCTCCACGCCTATGTTGTTGTTTTCGTTGTCTCAGGCGTACTCGTGCACCAGTTATTGACCAGACCGTTTTTGCTGTTGCGCGCGTGCCGTGTGCCGCTTGTCAATTGGAACCAGTC |
| 12 | BstXI | GCAAATGGCATTCTGACATCCACCACCACCCCCTCCTTGTTGTGGCGCACCACTTCGAGCCGCGAATCGCCGGCTCACGCCCTCTGGCACCTTCAATTTGGGCACCGGGTAGGGCGAGACGAGCGGCGTCCTCATGTACTCCCAtttggtTGGCGGCCTTTTTAAAGACCGTAAAGAAAAATAAGCACAAGTTTTATCCGGCCTTTATTCACATTCTTGCCCGCCTGATGAATGCTCATCCGGAgTTCCGTATGGCAATGCCAaggaaaTGGCTCGTTGTCTGGCCCATACAGATCCACCACCTCTGCCGCTACATTTGCTTCTCCTCCCGCTCTGCTCGATTCCTCCCCTTGCTGCATTTCGATTATTTTGGAAAAGAAAAGAAAGAAAGAGCTTGTCAATTGGAACCAGTC |
| 13 | BstXI | GCAAATGGCATTCTGACATCCAATAAAAGTTTGTTGCTAATCGATGCCACGCAAACAGGGAGGCAAGTGAGGAGGAATTGAACCGAACGTGGTAACGAGTGGAGAGGGAGACAAAAATGAGTGAATCGGTGTGGGGACGATCCAttgggtTGGCGGCCTTTTTAAAGACCGTAAAGAAAAATAAGCACAAGTTTTATCCGGCCTTTATTCACATTCTTGCCCGCCTGATGAATGCTCATCCGGAgTTCCGTATGGCAATGCCAagggaaTGGAAGATCAAGAGACACATGAAGATCAAGGCCTTAATGGAGCACAACAGACACATCACCGCCACCCAGCCAGCCTTGAAGATTCAGACGAAGCCTTCTTCGGTGAGGAAGATGGGCTCGGGGGCTTGTCAATTGGAACCAGTC |
| 14 | BstXI | GCAAATGGCATTCTGACATCCatgccagcaattatgacaatgttagcagaccatgcagctcgtcagctgcttgatttcagccaaaaactggatatcaacttattagataatgtggtgaattgcttataccatggagaaggaCCAttggttTGGCGGCCTTTTTAAAGACCGTAAAGAAAAATAAGCACAAGTTTTATCCGGCCTTTATTCACATTCTTGCCCGCCTGATGAATGCTCATCCGGAgTTCCGTATGGCAATGCCAaaggaaTGGggaataaaaaaatacgttgttggcctcattatcaagacgtcatctgacccaacttgtgtagagaaagaaaaggtgtatatcggaaaattaaatatgatccttgttcagatactgaaacaaGCTTGTCAATTGGAACCAGTC |
| 15 | BpiI | GCAAATGGCATTCTGACATCCgaattctcacagatatttcaactgtgtcagtttgtaatggaaaattctcaaaatgctccacttgtacatgcaaccttggaaacattgctcagatttctgaactggattcccctgggatatGAAGACaaaaacCGGCCTTTTTAAAGACCGTAAAGAAAAATAAGCACAAGTTTTATCCGGCCTTTATTCACATTCTTGCCCGCCTGATGAATGCTCATCCGGAgTTCCGTATGGCAATGGAAGACaactttaataccaatattcgacttgcgtactcaaatggaaaagatgatgaacagaacttcattcaaaatctcagtttgtttctctgcacctttcttaaggaacatgatcaacttatagaaaaaagaGCTTGTCAATTGGAACCAGTC |
| 16 | BpiI | GCAAATGGCATTCTGACATCCTTTAGCCTCGCGCTGAACGTATGCTGGTCGCGCGTGTGTCGTGTCGTTGGTCGTGTCGTGTCATGCCGTGTCCGTCTAGCTGGACCGGGAAACGTGGGAGCCCCTTTGCCTGCCCGCTCTGAAGACaaaaccCGGCCTTTTTAAAGACCGTAAAGAAAAATAAGCACAAGTTTTATCCGGCCTTTATTCACATTCTTGCCCGCCTGATGAATGCTCATCCGGAgTTCCGTATGGCAATGGAAGACaaccttCCCAGCATAGATTCCCTGCAGGGCCTGTCGGAGATTGAGGCCGAGAAGAGTGCGCTCACGCTCCTTTACACTCCGTGCTCTTGTCACTTGGCCTCACGCCATCCCTGTGCACACATGTGTGCTTGTCAATTGGAACCAGTC |
| 17 | BpiI | GCAAATGGCATTCTGACATCCGACGGCCAACAGAAGAGCTCTCCACTCTATTGAACCAAACACAGAAAGAGAACGACCGGCTCAAGCAGCGCGTGGCTCTCCTCTCTGCAGAGAAGAAGGTAGGCTCCCACCTCTCTCTCTGAAGACaaacccCGGCCTTTTTAAAGACCGTAAAGAAAAATAAGCACAAGTTTTATCCGGCCTTTATTCACATTCTTGCCCGCCTGATGAATGCTCATCCGGAgTTCCGTATGGCAATGGAAGACaaccctCTCTCTCTCTCTCGCTTCACGCTTATCGGTTGGTGGTACCTCGTCACACGCACCACTGCTTACCACACACACGCACGCACACACGACGCAATCAGACGGATTCGGGCGCCGAGATTGCGAGCTTGTCAATTGGAACCAGTC |
| 18 | BpiI | GCAAATGGCATTCTGACATCCCTCTGCAGGCGCAGCACCAGGAGCGACTGCAAAAGATCGTGGAGGCACACAATCAGGACCTCACGCTCCTGCAGGAGACCTACCAGGCGTGCGCTGCGCGACTCAAGGATACCGACGCGGGAAGACaaaccaCGGCCTTTTTAAAGACCGTAAAGAAAAATAAGCACAAGTTTTATCCGGCCTTTATTCACATTCTTGCCCGCCTGATGAATGCTCATCCGGAgTTCCGTATGGCAATGGAAGACaatcctCCTTGGCCGAGGAGAAGAGAAGGGTGGCCGACCTCGAGCGGGCGCGACAAGACCTCGCTGCTGCCCAACTGGCCGCGAAACCAGCGCCTCCACCATCGACCGACGTGCCAACCGACGTGCGCTTGTCAATTGGAACCAGTC |
