# Supplementary material for: SAMHD1 restrains aberrant nucleotide insertions at repair junctions generated by DNA end joining
Source: Nucleic Acids Res. 2021 Feb 16;49(5):2598–608. doi: 10.1093/nar/gkab051 (PMC7969033; doi:10.1093/nar/gkab051)
Supplement: gkab051_Supplemental_Files [file gkab051_supplemental_files.zip › Table S2.docx]

Sanger sequences from supporting Figure S3

Sample: HEK293 SAMHD1 KO

Length seq

156 GGCCGGGGGGAGCTTGCCACCCTGAAGGGAAGGGCACAAGCCTGGCTGGATTTCCCATCTGCTGACTGAAGAGCC-DSB- TGGGCAACATGGTGAAACCCCATGTCTACTAAAAATACAAAACTTAGCTGGGCGTGGGGCCACATGCTTTTGTAATCCCAG chr11: 106992749 - 107662545

338 GGCCGGGGGGAGCTTGCCACCCTGGCGTGCAGCTCGCCCAGGTGGATCTGGTGGGGGATGCTGCCGTTGTCGAAGGTGCGCTGCTTGCGCAGCAGGTCCTCGCGGTTCAGCTTCACCAGCAGCTCCTCGGTGCCGTCCATCTTCTCCAGGATGGGCTTGATGAACTTGTAGAACTCCTCCTGGCTGGCGCCGCCGTCGATGTAGCCGGCGTAGCCGTTCTTGCTCTGGTCGAAGAAGATCTCCTTGTACTTCTCGGGCAGCTGCTGGCGCACCAGGACCTGTCTACTAAAAATACAAAACTTAGCTGGGCGTGGGGCCACATGCTTTTGTAATCCCAG Cas9

269 GGCCGGGGGGAGCTTGCCACCCTGAAGGGAAGGGCACAAGCCTGGCTGGATTTCCCATCTGCTGACTGAAGAGCC TGGATTTCCCATCTGCTGACTGAAGAGCC TGGCTGGGTGTGGTGGCGCATGCCTGTAGTCCCAGCTACTCAGGAGGCTGAGGCAGGAGAATCGCTTGAACCCAGGAGGCGGAATGGGCAACATGGTGAAACCCCATGTCTACTAAAAATACAAAACTTAGCTGGGCGTGGGGCCACATGCTTTTGTAATCCCAG (GATAD2B) chr1:153848563-153848638

245 GGCCGGGGGGAGCTTGCCACCCTGAAGGGAAGGGCACAAGCCTGGCTGGATTTCCCATCTGCTGACTG TGGGCAACATGGTGAAACCCCATGTCTACTAAAAATACAAAACTTAGCTGGGC AGGGAAGGGCACAAGCCTGGCTGGATTTCCCATCTGCTGACTGTGGGCAACATGGTGAAACCCCATGTCTACTAAAAATACAAAACTTAGCTGGGCGTGGGGCCACATGCTTTTGTAATCCCAG

211 GGCCGGGGGGAGCTTGCCACCCTGAAGGGAAGGGCACAAGCCTGGCTGGATTTCCCATCTGCTGACTGAAGAGCC AGTGGGCAACATGGTGAAACCCCATGTCTACTAAAAATACAAAACTTAGCTGGGCTGGGCAACATGGTGAAACCCCATGTCTACTAAAAATACAAAACTTAGCTGGGCGTGGGGCCACATGCTTTTGTAATCCCAG

205 GGCCGGGGGGAGCTTGCCACCCTGAAGGGAAGGGCACAAGCCTGGCTGGATTTCCCATCTGCTGACTGAAGAGC ATGTCTACTAAAAATACAAAACTTAGCTGGGC ACAAGCCTGGCTGGATTTCCCATCTGCTGACTGAAGAGCATGTCTACTAAAAATACAAAACTTAGCTGGGCGTGGGGCCACATGCTTTTGTAATCCCAG

196 GGCCGGGGGGAGCTTGCCACCCTGAAGGGAAGGGCACAAGCCTGGCTGGATTTCCCATCTGCTGACTGAAGAGC GAAGGGCACAAGCCTGGC TGGATTTCCCATCTGCTGACTGATGGGCAACATGGTGAAACCCCATGTCTACTAAAAATACAAAACTTAGCTGGGCGTGGGGCCACATGCTTTTGTAATCCCAG

185 GGCCGGGGGGAGCTTGCCACCCTGAAGGGAAGGGCACAAGC TGGGCAACATGGTGAAACCCCATGTCTACTAAAAATACAAAACTTAG AGGGAAGGGCACAAGCTGGGCAACATGGTGAAACCCCATGTCTACTAAAAATACAAAACTTAGCTGGGCGTGGGGCCACATGCTTTTGTAATCCCAG

174 GGCCGGGGGGAGCTTGCCAC AGGGAAGGGCACAAGCCTGGCTGGATTTCCCATCTG GCCTGGCTGGATTTCCCATCTGCTGACTGAAGAGCCTTGGGCAACATGGTGAAACCCCATGTCTACTAAAAATACAAAACTTAGCTGGGCGTGGGGCCACATGCTTTTGTAATCCCAG

173 GGCCGGGGGGAGCTTGCCACCCTGAAGGGAAGGGCACAAGCCTGGCTGGATTTCCCATCTGCTG CTCTTGGGCAACATGGTGAAACCCCATGTCTACTAAAAATACAAACCCCATGTCTACTAAAAATACAAAACTTAGCTGGGCGTGGGGCCACATGCTTTTGTAATCCCAG

152 GGCCGGGGGGAGCTTGCCACCCTGAAGGGAAGGGCACAAGCCTGGCTGGATTTCCCATCTGCTGACTGAAGAGCC TGCACGAGCACATCGCCAACCTGGCCGGCAGCCCCGCCATCAAGAGGGCGTGGGGCCACATGCTTTTGTAATCCCAG Cas9

147 GGCCGGGGGGAGCTTGCCACCCTGAAGGGAAGGGCACAAGCCTGGCTGGATTTCCCATCTGCTGACTG GGCAACATGGTGAAACCCCATGTCTACTAAAAATACAAAACTTAGCTGGGCGTGGGGCCACATGCTTTTGTAATCCCAG

142 GGCCGGGGGGAGCTTGCCACCCTGAAGGGAAGGGCACAAGCCTGGCTGGATTTCCCATCTG TGGGCAACATGGTGAAACCCCATGTCTACTAAAAATACAAAACTTAGCTGGGCGTGGGGCCACATGCTTTTGTAATCCCAG

113 GGCCGGGGGGAGCTTGCCACCCTGAAGGGAAGGGCACAAGCCTGGCTGGATTTCCCATCTGCTGACTGAAGA AACTTAGCTGGGCGTGGGGCCACATGCTTTTGTAATCCCAG

105 GGCCGGGGGGAGCTTGCCACCCTGAAGGCAA CATGGTGAAACCCCATGTCTACTAAAAATACAAAACTTAGCTGGGCGTGGGGCCACATGCTTTTGTAATCCCAG

94 GGCCGGGGGGAGCTTGCCACCCTGA TGAAACCCCATGTCTACTAAAAATACAAAACTTAGCTGGGCGTGGGGCCACATGCTTTTGTAATCCCAG

Sample: HEK293 SAMHD1 KO + overexpressed wt-SAMHD1

156 GGCCGGGGGGAGCTTGCCACCCTGAAGGGAAGGGCACAAGCCTGGCTGGATTTCCCATCTGCTGACTGAAGAGCC-DSB-TGGGCAACATGGTGAAACCCCATGTCTACTAAAAATACAAAACTTAGCTGGGCGTGGGGCCACATGCTTTTGTAATCCCAG

140 GGCCGGGGGGAGCTTGCCACCCTGAAGGGAGGGAAGGGCACAAGCCTGGCTGGATTTCCCATCTGCTGA GGTGAAACCCCATGTCTACTAAAAATACAAAACTTAGCTGGGCGTGGGGCCACATGCTTTTGTAATCCCAG

116 GGCCGGGGGGAGCTTGCCACCCTGAAGGGAAGGGCACAAGCCTGGCTGGATTTCCCAT GTCTACTAAAAATACAAAACTTAGCTGGGCGTGGGGCCACATGCTTTTGTAATCCCAG

109 GGCCGGGGGGAGCTTGCCACCCTGAAGGGAAGGGCACAAGCCTGGCTGGAT GTCTACTAAAAATACAAAACTTAGCTGGGCGTGGGGCCACATGCTTTTGTAATCCCAG

103 GGCCGGGGGGAGCTTGCCACCCTGAAGGGAAGGG TGAAACCCCATGTCTACTAAAAATACAAAACTTAGCTGGGCGTGGGGCCACATGCTTTTGTAATCCCAG

98 GGCCGGGGGGAGCTTGCCACCCTGAAGGGA GAAACCCCATGTCTACTAAAAATACAAAACTTAGCTGGGCGTGGGGCCACATGCTTTTGTAATCCCAG

98 GGCCGGGGGGAGCTTGCCACCCTGAAGGGAAGGGCACAAGCCTGGCTGGA AATACAAAACTTAGCTGGGCGTGGGGCCACATGCTTTTGTAATCCCAG

96 GGCCGGGGGGAGCTTGCCACCCTGAAGGGAAGG CCCATGTCTACTAAAAATACAAAACTTAGCTGGGCGTGGGGCCACATGCTTTTGTAATCCCAG

94 GGCCGGGGGGAGCTTGCCACCCTGAAGG AACCCCATGTCTACTAAAAATACAAAACTTAGCTGGGCGTGGGGCCACATGCTTTTGTAATCCCAG

93 GGCCGGGGGGAGCTTGCCACCCTGAAGGG CACCATGTCTACTAAAAATACAAAACTTAGCTGGGCGTGGGGCCACATGCTTTTGTAATCCCAG

78 GGCCGGGGGGAGCTTGCCA TGTCTACTAAAAATACAAAACTTAGCTGGGCGTGGGGCCACATGCTTTTGTAATCCCAG

75 GGCCGGGGGGAGCTTGCCACCCTGAAGGGAAG

A CATGTCTA CTGGGCGTGGGGCCACATGCTTTTGTAATCCCAG

74 GGCCGGGGGGAGCTTGCCACCCTGAAGGGAAGGGCACAAGCCTGGC GTGGGGCCACATGCTTTTGTAATCCCAG

74 GGCCGGGGGGAGCTTGCCACCCTGAAGGGAAGGGCACAAGCCTGGCGTGG GGCCACATGCTTTTGTAATCCCAG

Sample: HEK293 SAMHD1 KO + K312A

156 GGCCGGGGGGAGCTTGCCACCCTGAAGGGAAGGGCACAAGCCTGGCTGGATTTCCCATCTGCTGACTGAAGAGCC-DSB- TGGGCAACATGGTGAAACCCCATGTCTACTAAAAATACAAAACTTAGCTGGGCGTGGGGCCACATGCTTTTGTAATCCCAG

481 GGCCGGGGGGAGCTTGCCACCCTGAAGGGAAGGGCACAAGCCTGGCTGGATTTCCCATCTGCCCCCATGTCTACTAAAAATACAAAACTTA AGGGAAGGGCACAAGCCTGGCTGGATTTCCCATCTGC CCCCATGTCTACTAAAAATACAAAACTTAGCTGGCGGAATCGGCTCGCTGCATGG TACCAAGCTTGGTGGCGGCCTAGAATAGAATGACACCTACTCAGACAATGCGATGCAATTTCCTCATTTTATTAGGCGACGCAATCGTCCGATCCGGAGCCGGGACTGTCGGGCGTACACAAATCGCCCGCAGGGAAGGGCACAAGCCTGGCTGGATTTCCCATCTGCCCCCATGTCTACTAAAAATACAAAACTTAAGGGAAGGGCACAAGCCTGGCTGGATTTCCCATCTGCCCCCATGTCTACTAAAAATACAAAACTTAGCTGGGCGTGGGGCCACATGCTTTTGTAATCCCAG vector (samhd1 expression)

473 GGCCGGGGGGAGCTTGCCACCCTGAAGGGAAGGGCACAAGCCTGGCTGGATTTCCCATCTGCTGA AGTGGCACCTGACCGAGCACGCCATCGCCTCCGGCTCCGCCTTGCCCTGAGGGCCCAGATCTCCTGAGGCTGCAGTCTAGAGGATCATAATCAGCCATACCACATTTGTAGAGGTTTTACTTGCTTTAAAAAACCTCCCACACCTCCCCCTG ATGGACTATCATATGCTTACCGTAACTTGAAAGTATTTCGATTTCTTGGCTTTATATATCTTGTGGAAAGGACGAAACACCGGGGCTTGTGCCCTTCCCTTCAGTTTAAGAGCTATGCTGGAAACAGCATAGCAAGTTTAAATAAGGCTAGTCCGTTATCAACTTGAAAAAGTGGCGGGCAACATGGTGAAACCCCATGTCTACTAAAAATACAAAACTTAGCTGGGCGTGGGGCCACATGCTTTTGTAATCCCAG Cas vector; Cas Vector

420 GGCCGGGGGGAGCTTGCCACCCTGAAGGGAAGGGCACAAG ACATATACTCAGGTTTTAAAATAGGGTACAATCAAAATAGTGCTTTTCTCTAGACTAAGAATCCTCTAATTTTTTATTTATTTGTCTGGTTTTTGTCTGCAACTAGACATTCTTGAGAGCAGGAATATACTTCAATATTATACACCCAAGGCACGTAGTGCTATGCCACACAACAGAAACTCAATAAATAGTTCAACATAAAAATCTAGGGATAAGAAAAGCAGATTCCATAAACTAACATCAGACAAGTCCAAGAGAGAGTCAACAGTTCACATCAAAAAGAAAAGGAAGCAGTGATCTGGGCAACATGGTGAAACCCCATGTCTACTAAAAATACAAAACTTAGCTGGGCGTGGGGCCACATGCTTTTGTAATCCCAG (PHF3) chr6:64403627-64403925

398 GGCCGGGGGGAGCTTGCCACCCTGAAGGGAAGGGCA TCTGCTGACTGAAGGCCCTGGTGCGCCAGCAGCTGCCCGAGAAGTACAAGGAGATCTTCTTCGACCAGAGCAAGAACGGCTACGCCGGCTACATCGACGGCGGCGCCAGCCAGGAGGAGTTCTACAAGTTCATCAAGCCCATCCTGGAGAAGATGGACGGCACCGAGGAGCTGCTGGTGAAGCTGAACCGCGAGGACCTGCTGCGCAAGCAGCGCACCTTCGACAACGGCAGCATCCCCCACCAGATCCACCTGGGCGAGCTGCACGCCATCCTGCGCCGCTGGGCAACATGGTGAAACCCCATGTCTACTAAAAATACAAAACTTAGCTGGGCGTGGGGCCACATGCTTTTGTAATCCCAG vector

393 GGCCGGGGGGAGCTTGCCACCCTGAAGGGAAGGGCACAAGCCTGGCTGGATTTCCCATCTGCTGA TGGGCAACATGGTGAAACCCCATGTCTACTAAAAATACAAAACTTAGCTGGGCTGGGCGGCGGCAAAATGGTTCACGAAGTGAGTTTTGACGGCTTCACGACTCAATCTGCGCCGTGGAAACTGGAAGCTGGAACGCCAAATGTCGCTGGTGTCATAGGATTAAGCGCGGCGCTGGAATGGCTGGCAGATTACGATATCAACCAGGCCGAGAAGGGCACAAGCCTGGCTGGATTTCCCATCTGCTGATGGGCAACATGGTGAAACCCCATGTCTACTAAAAATACAAAACTTAGCTGGGCGTGGGGCCACATGCTTTTGTAATCCCAG no relevant homology (plasmid or human)

361 GGCCGGGGGGAGCTTGCCACCCTGAAGGCCCTGGCCCACATGATCAAGTTCCGCGGCCACTTCCTGATCGAGGGCGACCTGAACCCCGACAACAGCGGAGCATCAAGTCACATAAGAATGTGCTATGCTGAGGATTTCCCTAGCGGTCCAGTGCTTCTTCTCCAGGGGCCACAGGGATCTCGGGGTTGCATTCCAGACGCACCCGGAGAGACAGGCATTCATCTCGAGTGGATTGGGCAACATGGTGAAACCCCATGTCTACTAAAAATACAAAACTTAGCTGGGCC CATGGTGAAACCCCATGTCTACTAAAAATACAAAACTTAGCTGGGCGTGGGGCCACATGCTTTTGTAATCCCAG vector

361 GGCCGGGGGGAGCTTGCCACCCTGAAGGGAAGGGCACAAGCCTGGCTGGATTTCCCATCTGCTGACATAGTGTTCACATACAAGTTATTAACAATGACAAGACTACATCAACCACTCACAGCACACAAAACAAATTTCTACAAACCCTGGGGAGGGGGTGTCTTCGGGGTCTCTAAGGATAAATTAGTGCTCTTTAGTGTATCTGTAGGATGTTTCTGATGGACTACATTTTGCACAGGAGATGTGTTGTACCAACCGAAAATAAATCGACTAAGAGTTATTGTCCTAGGTCCTTGACCTTTTTTATC CTAAAAATACAAAACTTAGCTGGGCGTGGGGCCACATGCTTTTGTAATCCCAG (ZIC5) chr13:100616066-100616307

319 GGCCGGGGGGAGCTTGCCACCCTGAAGGGGACGGATGACACGATATATGTTTTAATAAGTGTTTAAAAATCACTGGTCTGAAAGAAGAAACTTTCAAATCTGTTGATGCCCTCCTCGATGCGCTTCATGCGCTCGCGGCTGTTCTTCTGGCCCTTCTGGGTGGTCTGGTTCTCGCGGGCCATCTCGATCACGATGTTCTCGGGCTTGTGGCGGCCCATCACCTTCACCAGCTCGTCCACCACCTTCAC GGTGAAACCCCATGTCTACTAAAAATACAAAACTTAGCTGGGCGTGGGGCCACATGCTTTTGTAATCCCAG Cas9

295 GGCCGGGGGGAGCTTGCCACCCTGAAGGGAAGGGCACAAGCCTGGCTGGATTTCCCATCTGCTGACTGAAGAGCCC TGCTTCAGCCTCCCAAGTAGCTGGGATTACAGGCACATGCCACCACGCCCAGCTAATTTTTTGTATTTTCAGTAGAGACAGGGTTTCACCATGTTGGATGGATTTCCTAGTCAAATGAGTTTTCGCCTCAGTGAGCTCAGGGCAACATGGTGAAACCCCATGTCTACTAAAAATACAAAACTTAGCTGGGCGTGGGGCCACATGCTTTTGTAATCCCAG (RAB3GAP2) chr1:220325529-220325624

289 GGCCGGGGGGAGCTTGCCACCCTGAAGGGAAGGGCACAAGCCTGGCTGGATTTCCCATC CTGCGCCGGTTGCATTCGATTCCTGTTTGTAATGGGCAACATGGTGAAACCCCATGTCTACTAAAAATACAAAACTTAGCTG AAGGGAAGGGCACAAGCCTGGCTGGATTTCCCATC CTGCGCCGGTTGCATTCGATTCCTGTTTGTAATGGGCAACATGGTGAAACCCCATGTCTACTAAAAATACAAAACTTAGCTGGGCGTGGGGCCACATGCTTTTGTAATCCCAG

193 GGCCGGGGGGAGCTTGCCACCCTGAAGGGAAGGGCACAAGCCTGGCTGGATTTCCCATCTGCTGACTGAAGAG TCTACTAAAAATACAA GGGAAGGGCACAAGCCTGGCTGGATTTCCCATCTGC TGACTGAAGAGTCTACTAAAAATACAAAACTTAGCTGGGCGTGGGGCCACATGCTTTTGTAATCCCAG

190 GGCCGGGGGGAGCTTGCCACCCTGAAGGGAAGGGCACAAGCCTGGCTGGATTTCCCATCTGCTGACTGAAGAGCC AGGGAAGGGCACAAGCCTGGCTGGATTTCCCATCTGGGCAACATGGTGAAACCCCATGTCTACTAAAAATACAAAACTTAGCTGGGCGTGGGGCCACATGCTTTTGTAATCCCAG

188 GGCCGGGGGGAGCTTGCCACCCTGAAGGGAAGGGCACAAGCCTGGCTGGATTTCCCATCTGCTGACTGAAGAGCC TGGGCAACATGGTGAAACCCCATGTCTACTAATGGGCAACATGGTGAAACCCCATGTCTACTAAAAATACAAAACTTAGCTGGGCGTGGGGCCACATGCTTTTGTAATCCCAG

179 GGCCGGGGGGAGCTTGCCACCCTGAAGGGAAGGGCACAAGCCTGGCTGGATTTCCCATCTGCTGACTGAAGAGCCTGGATTTCCCATCTGCTGACTGAAGAGCC GCATGGTGAAACCCCATGTCTACTAAAAATACAAAACTTAGCTGGGCGTGGGGCCACATGCTTTTGTAATCCCAG

153 GGCCGGGGGGAGCTTGCCACCCTGAAGGGAAGGGCACAAGCCTGGCTGGATTTCCCATCTGCTGACTGAAGA TGGGCAACATGGTGAAACCCCATGTCTACTAAAAATACAAAACTTAGCTGGGCGTGGGGCCACATGCTTTTGTAATCCCAG

148 GGCCGGGGGGAGCTTGCCACCCTGAAGGGAAGGGCACAAGCCTGGCTGGATTTCCCATCTGCTGAC ATGGGCAACATGGTGAAACCCCATGTCTACTAAAAATACAAAACTTAGCTGGGCGTGGGGCCACATGCTTTTGTAATCCCAG

136 GGCCGGGGGGAGCTTGCCACCCTGAAGGGAAGGGCACAAGCCTGGCTGGATTTCCCATC AGTCAG GGTGAAACCCCATGTCTACTAAAAATACAAAACTTAGCTGGGCGTGGGGCCACATGCTTTTGTAATCCCAG

131 GGCCGGGGGGAGCTTGCCACCCTGAAGGGAAGGGCACAAGCCTGGCTGGATTTCCCATCTGCTGA AACCCCATGTCTACTAAAAATACAAAACTTAGCTGGGCGTGGGGCCACATGCTTTTGTAATCCCAG

125 GGCCGGGGGGAGCTTGCCACCCTGAAGGGAAGGGCACA TGGATT TGGGCAACATGGTGAAACCCCATGTCTACTAAAAATACAAAACTTAGCTGGGCGTGGGGCCACATGCTTTTGTAATCCCAG

115 GGCCGGGGGGAGCTTGCCACCCTGAAGGGAAGGG TGGGCAACATGGTGAAACCCCATGTCTACTAAAAATACAAAACTTAGCTGGGCGTGGGGCCACATGCTTTTGTAATCCCAG

112 GGCCGGGGGGAGCTTGCCACCCTGAAGGAAA TGGGCAACATGGTGAAACCCCATGTCTACTAAAAATACAAAACTTAGCTGGGCGTGGGGCCACATGCTTTTGTAATCCCAG

107 GGCCGGGGGGAGCTTGCCACCCTGAA TGGGCAACATGGTGAAACCCCATCTCTACTAAAAATACAAAACTTAGCTGGGCGTGGGGCCACATGCTTTTGTAATCCCAG

104 GGCCGGGGGGAGCTTGCCACCCTGAAG CAACATGGTGAAACCCCATGTCTACTAAAAATACAAAACTTAGCTGGGCGTGGGGCCACATGCTTTTGTAATCCCAG

102 GGCCGGGGGGAGCTTGCCACCCTGAAGGGAAGGGCACAAGCCTGGCTGGATTTCCCATCTGCTGACTGAAGAGC GTGGGGCCACATGCTTTTGTAATCCCAG

95 GGCCGGGGGGAGCTTG GGCAACATGGTGAAACCCCATGTCTACTAAAAATACAAAACTTAGCTGGGCGTGGGGCCACATGCTTTTGTAATCCCAG

94 GGCCGGGGGGAGCTTGCCACCCTGAAGGG TCCCCATGTCTACTAAAAATACAAAACTTAGCTGGGCGTGGGGCCACATGCTTTTGTAATCCCAG

Sample: HEK293 WT

156 GGCCGGGGGGAGCTTGCCACCCTGAAGGGAAGGGCACAAGCCTGGCTGGATTTCCCATCTGCTGACTGAAGAGCC-DSB- TGGGCAACATGGTGAAACCCCATGTCTACTAAAAATACAAAACTTAGCTGGGCGTGGGGCCACATGCTTTTGTAATCCCAG

212 GGCCGGGGGGAGCTTGCCACCCTGAAGGGAAGGGCACAAGCCTGGCTGGATTTCCCATCTGCTGACTGAAGAGCCAGGGCACAAGCCTGGCTGGATTTCCCATCTGCTGACTGAAGAGCC TGGCTGGATTTCCC ATCTGCTGACTGAAGAGCC AGGGCACAAGCCTGGCTGGATTTCCCTGGGCGTGGGGCCACATGCTTTTGTAATCCCAG

206 GGCCGGGGGGAGCTTGCCACCCTGAAGGGAAGGGCACAAGCCTGGCTGGA AAACATGGTGAAACCCCATGTCTATGGGCAACATGGTGAAACCCCATGTC GCAACATGGTGAAACCCCATGTCTATGGGCAACATGGTGAAACCCCATGTCTACTAAAAATACAAAACTTAGCTGGGCGTGGGGCCACATGCTTTTGTAATCCCAG

194 GGCCGGGGGGAGCTTGCCACCCTGAAGGGAAGGGCACAAGCCTGGCTGGATTTCCCATCTGCTGACTGAAGAGCC GGGAAGGGCACAAGCCTGGCTGGATTTCCCATCTGCTGACTGAAGCATGGTGAAACCCCATGTCTACTAAAAATACAAAACTTAGCTGGGCGTGGGGCCACATGCTTTTGTAATCCCAG

175 GGCCGGGGGGAGCTTGCCACCCTGAAGGGA CGGCCGCCTGAGCCGCAAGCTTATCAACGGCATCCGCGACAAGCAGAGCGGCAAGACCATCCTATGGGCAACATGGTGAAACCCCATGTCTACTAAAAATACAAAACTTAGCTGGGCGTGGGGCCACATGCTTTTGTAATCCCAG Cas9

170 GGCCGGGGGGAGCTTGCCACCCTGAAGGGAAGGGCACAAGCCTGGCTGGATTTCCCATCTGCTGGGAAGGGCACAAGCCTGGCTGGATTTCCCATCTGCTGACTGAAG CCATGTCTACTAAAAATACAAAACTTAGCTGGGCGTGGGGCCACATGCTTTTGTAATCCCAG

142 GGCCGGGGGGAGCTTGCCACCCTGAAGGGAAGGGCACAAGCCTGGCTGGATTTCCCATCTGCTGACTG CATGGTGAAACCCCATGTCTACTAAAAATACAAAACTTAGCTGGGCGTGGGGCCACATGCTTTTGTAATCCCAG

138 GGCCGGGGGGAGCTTGCCACCCTGAAGGGAAGGGCACAAGCCTGGCTGGATTTCCCAT GGGCAACATGGTGAAACCCCATGTCTACTAAAAATACAAAACTTAGCTGGGCGTGGGGCCACATGCTTTTGTAATCCCAG

131 GGCCGGGGGGAGCTTGCCACCCTGAAGGGAAGGGCA TGGGCAACATGGTGAAACCCCATGTCTACTACCCCATGTCTACTAAAAATACAAAACTTAGCTGGGCGTGGGGCCACATGCTTTTGTAATCCCAG

130 GGCCGGGGGGAGCTTGCCACCCTGAAGGGAAGGG GTGAAACCCCATGTCTACTAAAAATACAAAAACCCCATGTCTACTAAAAATACAAAACTTAGCTGGGCGTGGGGCCACATGCTTTTGTAATCCCAG

130 GGCCGGGGGGAGCTTGCCACCCTGAAGGGAAGGGCACAAGCCTGGCTGGATTTCCC CATGGTGAAACCCCATGTCTACTAAAAATACAAAACTTAGCTGGGCGTGGGGCCACATGCTTTTGTAATCCCAG

120 GGCCGGGGGGAGCTTGCCACCCTGAAGGGAAGGGCACAA TGGGCAACATGGTGAAACCCCATGTCTACTAAAAATACAAAACTTAGCTGGGCGTGGGGCCACATGCTTTTGTAATCCCAG

113 GGCCGGGGGGAGCTTGCCACCCTGAAGGGAAGGGCACTAA ATGGTGAAACCCCATGTCTACTAAAAATACAAAACTTAGCTGGGCGTGGGGCCACATGCTTTTGTAATCCCAG

113 GGCCGGGGGGAGCTTGCCACCCTGAAGGGAAGGGC GCAACATGGTGAAACCCCATGTCTACTAAAAATACAAAACTTAGCTGGGCGTGGGGCCACATGCTTTTGTAATCCCAG

107 GGCCGGGGGGAGCTTGCCACCCTGAATGG GCAACATGGTGAAACCCCATGTCTACTAAAAATACAAAACTTAGCTGGGCGTGGGGCCACATGCTTTTGTAATCCCAG

102 GGCCGGGGGGAGCTTGCCACCCTGAAGGGA TGGTGAAACCCCATGTCTACTAAAAATACAAAACTTAGCTGGGCGTGGGGCCACATGCTTTTGTAATCCCAG

98 GGCCGGGGGGAGCTTGCCACCCTGAAGGGAAGGGCACAA TGTCTACTAAAAATACAAAACTTAGCTGGGCGTGGGGCCACATGCTTTTGTAATCCCAG

98 GGCCGGGGGGAGCTTGCCACCCTGAA TGGTGAAACCCCATGTCTACTAAAAATACAAAACTTAGCTGGGCGTGGGGCCACATGCTTTTGTAATCCCAG

95 GGCCGGGGGGAGCTTGCCACCCTGAAGGGAAG ACCATGTCTACTAAAAATACAAAACTTAGCTGGGCGTGGGGCCACATGCTTTTGTAATCCCAG

90 GGCCGGGGGGAGCTTGCCACCCTGAA CCCCATGTCTACTAAAAATACAAAACTTAGCTGGGCGTGGGGCCACATGCTTTTGTAATCCCAG

Legend:

Red letters: genomic DNA DSB (overhangs underlined)

Shaded blue: duplications

Shaded red: insertions

Shaded green: microhomologies

Length of depicted sequences indicated at the beginning of the sequence (length of joints from refilled overhangs: 156nt)

Relevant homologies of insertions to genomic sites indicated at the end of sequences
